# The Synthetic Extracellular Matrix as a Maestro of the In Vitro Stem Cell Niche: Orchestrating Fate and Function

**DOI:** 10.3390/biomedicines14020485

**Published:** 2026-02-23

**Authors:** Subhajit Giri, Pratyush Rajesh

**Affiliations:** 1 Department of Pathology, Medical College of Wisconsin, 8701 W Watertown Plank Rd., Milwaukee, WI 53226, USA; 2 Medical College of Wisconsin Cancer Center, 8701 W Watertown Plank Rd., Milwaukee, WI 53226, USA; 3 Department of Biology, Johns Hopkins University, Mudd Hall 144, 3400 N Charles St., Baltimore, MD 21218, USA; pratyush.rajesh@gmail.com

**Keywords:** synthetic extracellular matrix, Matrigel, human pluripotent stem cell, organoid, scaffold, differentiation, new approach methodologies

## Abstract

Human-induced pluripotent stem cells (hiPSCs) have an innate ability to differentiate into the three germ layers: the ectoderm, endoderm, and mesoderm. By using targeted differentiation methods and carefully controlling growth factors, morphogens, and signaling modulators, hiPSCs can be guided to develop into specific lineage cell types. For clinical applications of hiPSCs and their derivatives, it is crucial to use xenogen-free, chemically defined culture media, reagents, recombinant growth factors, morphogens, and extracellular matrix (ECM) scaffolds. One major obstacle is the widespread use of Matrigel as an hiPSC culture matrix. Matrigel, derived from Engelbreth–Holm–Swarm (EHS) mouse sarcoma, is an extract of basement membrane material with a complex, poorly defined, and variable composition. It also exhibits batch-to-batch variability in mechanical and biochemical properties and is difficult to modify, which limits its rational use in the production of therapeutic cells and organoids. Synthetic ECM matrices and scaffolds offer a promising alternative because they can have a fully defined composition, highly tunable physical properties, surface modifications, and functionalization with recombinant signaling peptides and growth factors. This provides a suitable microenvironment for hiPSC culture and the directed differentiation towards lineage-specific cells and organoid development, and can be used in clinical-grade tissue transplantation and regenerative medicine.

## 1. Introduction

The extracellular matrix (ECM) is an intricate network of fibrous proteins, proteoglycans, glycoproteins, and glycosaminoglycans that surrounds cells and serves as an anatomical scaffold, providing critical physical information that controls cellular behavior [1]. The ECM serves as a microenvironment rich in dynamic feedback signaling that regulates stem cell identity and function. The composition, organization, and stiffness of the ECM contribute to biological processes, including adhesion, migration, and differentiation [2]. The ECM consists of complexes of bioactive molecules such as growth factors, cytokines, adhesion ligands, and other signaling molecules, all of which interact with specific stem cell receptors and initiate downstream signaling pathways to modulate many of these cellular processes [3]. For instance, integrins are cell surface receptors for the ECM, such as fibronectin and laminin, and play a crucial role in regulating stem cell proliferation, differentiation, and survival [4,5]. ECM elasticity and stiffness also direct stem cell differentiation and fate determination. It has been shown that softer matrices increase the self-renewal capacity of mesenchymal stem cells (MSCs), whereas stiffer ECM scaffolds favor differentiation towards osteoblast lineages [6,7]. The ECM also acts as a depot for signaling molecules, such as growth factors and morphogens, which are sequestered and poised for release via enzymatic degradation or cell-mediated interactions, thereby generating local gradients that control stem cell migration, proliferation, and differentiation within the niche. The ECM is a dynamic entity rather than a static structure, constantly remodeled by resident cells. Different types of proteases secreted by stem cells can degrade and reorganize the surrounding ECM, altering niche composition and influencing stem cell fate and the behavior of neighboring cells. This dynamic interplay between stem cells and the ECM creates a self-regulating microenvironment that orchestrates stem cell function [8,9].

Understanding the intricate interplay between the ECM and stem cells holds immense potential for regenerative medicine. To serve this purpose, researchers can engineer synthetic ECMs. A synthetic ECM is an engineered biomaterial designed to mimic the structural, biochemical, mechanical, and functional properties of the native ECMs, while offering a precise control over composition, reproducibility, and reduced batch-to-batch variability [10]. Unlike natural ECMs, synthetic ECMs are typically composed of polymers, self-assembling peptides, biodegradable crosslinkers, and bioactive motifs, which enables tunable stiffness, degradability, and ligand presentation [11]. These matrices support cell adhesion, migration, proliferation, and differentiation with suitable physiochemical cues to the cells. Additionally, the ability to manipulate the ECM composition and signaling cues can potentially guide stem cell differentiation toward desired cell types for tissue repair and regeneration.

A tremendous number of efforts are underway to develop advanced bioinspired synthetic ECM and biomaterials to revolutionize regenerative medicine and stem cell-based therapeutic opportunities. Accumulating evidence suggests that the synthetic ECM can effectively replace the usage of Matrigel, a widely used ECM in stem cell and organoid-based research. At this inflection point, it is essential to keep track of the new inventions and knowledge base in the synthetic ECM and advanced engineered biomaterials domain. To help with this, a periodic collection of new information could be beneficial for research and for the therapeutic development community, which we tried to deliver in this review article.

## 2. Limitations of Matrigel as an Organoid Scaffold

**Figure 1:**
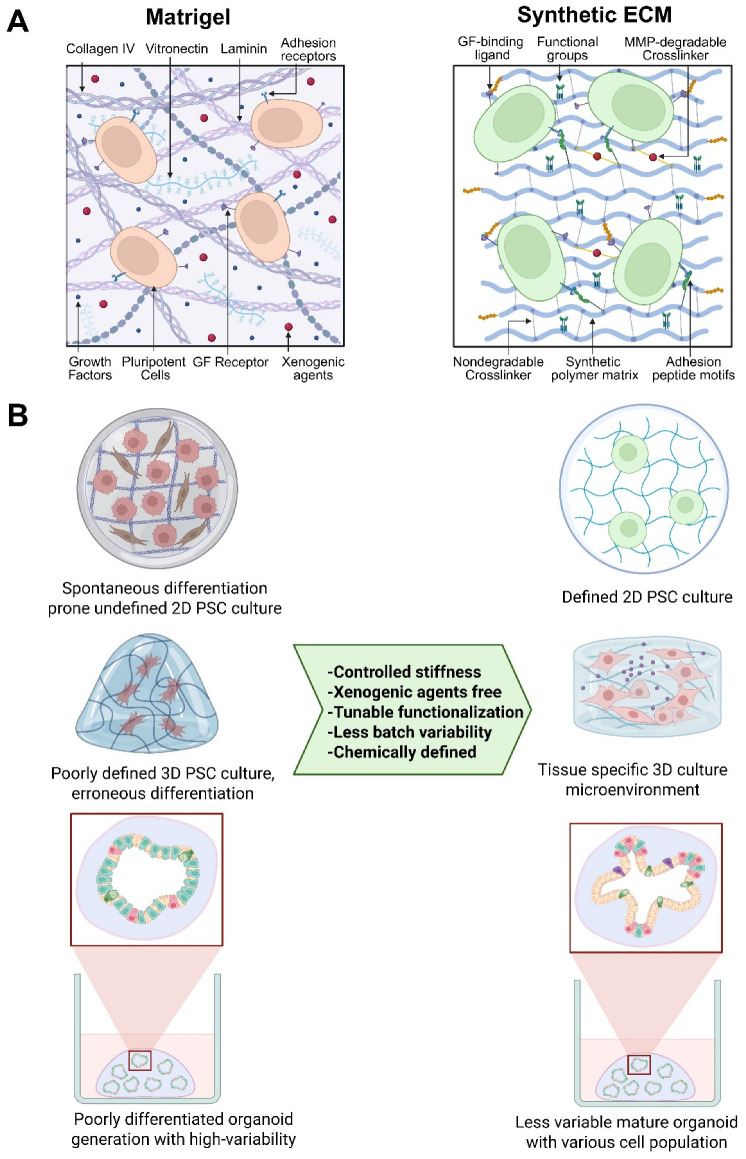
Structural and functional comparison of Matrigel and synthetic ECM scaffolds. (**A**) The left panel illustrates the major structural components of Matrigel, and the right panel of synthetic ECM scaffolds. In synthetic ECM, the distribution of growth factor-binding ligands, functional group modifications, matrix metalloprotease (MMP)-degradable crosslinkers, and adhesion peptide motifs can be well defined, tunable, and spatially engineered to enhance cell adhesion, proliferation, and controlled differentiation. While Matrigel lacks these properties, it also contains numerous undefined components, xenogenic factors, and pathogenic factors. (**B**) The illustration depicts the suitability of synthetic ECM for hPSC culture and differentiation to 2D cell types or 3D organoids. Created in BioRender. Giri, S. (2026) https://BioRender.com/7fxzl37 (accessed on 3 February 2026).

Matrigel was first invented by Prof. Hynda Kleinman [12,13]. In the early stages of 3D cell culture research, ‘Matrigel’ was a gold-standard ECM for in vitro studies, revolutionizing stem cell and organoid-based research. However, its application in cell biology research and therapeutic cell manufacturing is hindered by its complexity and batch-to-batch compositional variability. Numerous proteomic profiling and protein content analysis efforts identified more than 14,000 unique peptides and 2000 proteins in Matrigel [12,13,14,15,16,17,18]. Most of these proteins are structural proteins, growth factors, transcription factors, and cytokines [15,19]. Such growth factors and cytokines in the basement membrane could alter the characteristics of cultured stem cells and interfere with the developmental trajectory during directed differentiation. Growth factor reduced (GFR) Matrigel, which is structurally similar to standard Matrigel but contains fewer and lower concentrations of growth factors, could be an alternative to address this problem [14,19]. Another significant drawback is batch-to-batch variability, which introduces additional variability into experimental results. It has been shown that insulin-like growth factor 1 and epidermal growth factor were detected at nanogram-per-milliliter levels in a single batch of Matrigel [19]. In a later study, these growth factors were not detected in four independent Matrigel batches [15]. As summarized in Figure 1A, such inconsistency in key signaling molecules, including fibroblast growth factor 2 (FGF2) and Platelet-derived growth factors (PDGFs), has been reported across multiple batches of Matrigel [15].

Matrix viscoelasticity is a crucial mechanical cue for stem cell differentiation and organoid development [6,20,21]. The mechanical properties and stiffness of Matrigel were also inconsistent, as found in batch-to-batch and intra-batch variability [22,23]. Using atomic force microscopy, the elastic modulus (‘stiffness’) of two batches of Matrigel was reported to be 400–420 Pa, whereas another batch of Matrigel was 840 Pa [24]. Heterogeneity in local stiffness in Matrigel was also reported, where in situ mechanical interferometry identified a higher elastic modulus (1–3 kPa) [23,24]. Such inconsistency of Matrigel mechanical properties can impede proper stem cell culture and differentiation, organoid development, and tissue organization [20,25,26]. Another major drawback of Matrigel is the antigenicity and the presence of xenogenic contaminations, as it is an animal-derived ECM. Viral contamination, especially the lactate dehydrogenase-elevating virus (LDHV), has been found in multiple batches of Matrigel [27,28]. LDHV is a natural mouse virus that infects macrophages and can elicit immunogenic reactions and modulate tumor behavior [29,30]. The presence of such contaminants can impede mechanistic studies in cell biology experiments and hinder the interpretation of the underlying biological events. For these reasons, the usage of Matrigel in clinical applications and therapeutic cell therapy product development is limited. Using an animal-derived ECM, such as Matrigel, in human therapies carries the risk of zoonotic pathogens that can jeopardize patient safety. Matrigel cannot be fine-tuned to the microenvironment and the scaffold architecture required for stem cell differentiation and organoid culture, thereby limiting its use across a broader spectrum.

Geltrex (Gibco) and Cultrex (R&D Systems/Trevigen) are formulated as alternatives to Matrigel with higher laminin contents, though both are derived from EHS mouse sarcoma tumors. Both Geltrex and Cultrex provide robust support to hPSC pluripotency, efficient human intestinal organoid development, and reduced viscosity for easier pipetting [31]. Geltrex excels in soft embedding for long-term brain organoid maturation up to 150 days [32]. Still, both remain animal/xeno-derived and poorly defined ECMs, restricting their use in advanced translational research and clinical applications. They exhibit low mechanical strengths and rapid degradation, and limit large organoid (>1 mm) culture due to hypoxia and nutrient diffusion issues. Overall, Geltrex and Cultrex bridge gaps but underscore the need for fully defined matrices [33,34].

## 3. Comparison of Matrigel, Natural ECMs, and Synthetic ECMs

Matrigel, tissue-derived natural ECMs, and fully synthetic ECMs each play distinct and complementary roles in organoid research, and the most appropriate choice depends on the specific experimental goals and translational context. Matrigel remains the most widely used matrix for early-stage exploratory studies and initial organoid establishment. Its biochemical richness, including a broad array of growth factors such as FGF-2, VEGF, and TGF-β, has made it highly effective for supporting a wide range of organoids, including intestinal, gastric, hepatic, and neural systems [14,35]. However, Matrigel’s tumor-derived, xenogeneic origin, substantial batch-to-batch variability, high cost, and lack of GMP-compliant manufacturing severely limit its suitability for clinical translation and regenerative medicine [36,37].

Decellularized, tissue-derived ECMs offer a more physiologically relevant alternative by preserving native, tissue-specific biochemical cues and age-dependent ECM signatures. These matrices often support organoid development that matches or even exceeds that achieved with Matrigel, making them particularly valuable for optimizing differentiation protocols and modeling disease in contexts where native microenvironmental signaling is critical [38,39]. For example, gastrointestinal dECM hydrogels contain a richer repertoire of tissue-specific proteins than Matrigel, which has been linked to enhanced stemness, improved differentiation capacity, long-term culture stability, and successful organoid transplantation. Despite these advantages, natural ECMs still face regulatory hurdles and retain some degree of batch variability, even when produced under GMP-compatible conditions [38].

Fully synthetic ECMs are increasingly favored for mechanistic studies and translational applications that demand precision, reproducibility, and regulatory compliance. These systems allow independent and quantitative control over key matrix parameters, including stiffness, viscoelasticity, ligand density, and enzymatic degradability, while eliminating the confounding variability inherent to undefined natural matrices [35,40]. As a result, synthetic ECMs are particularly well suited for high-throughput drug screening, the systematic interrogation of ECM–cell interactions, and the development of xeno-free, GMP-compliant scaffolds for regenerative therapies (Figure 1B). Notably, certain polyethylene glycol (PEG)-based hydrogels have been shown to support human intestinal organoid formation, viability, and differentiation at levels comparable to, or exceeding, those achieved with Matrigel [41,42].

The main limitation of synthetic ECMs lies in their reduced biological complexity, as they lack the full spectrum of endogenous growth factors and cryptic binding sites present in natural matrices. However, this apparent drawback is also a strength. The modular design of synthetic systems allows researchers to precisely introduce defined adhesive ligands and protease-sensitive motifs, enabling mechanistic insights that are difficult to obtain in complex, ill-defined environments like Matrigel. For instance, synthetic matrices with independently tunable ligand spacing and stress–relaxation dynamics have revealed fundamental mechanotransduction principles that govern stem cell fate decisions, which we discuss elaborately later in Section 7. Emerging hybrid approaches that combine synthetic backbones with defined natural components aim to balance biological functionality with experimental control, potentially bridging the gap between discovery-driven research and clinical translation. The individual strengths and limitations of Matrigel, natural ECM, and synthetic ECM are consolidated in Table 1.

## 4. Synthetic ECM Scaffolds

The inherent limitations of Matrigel in stem cell and organoid-based applications have prompted the search for synthetic alternatives that can be fine-tuned with greater flexibility. In this context, numerous synthetic polymer-based 2D or 3D scaffolds have been developed. They can be modified through precise fabrication processes to control molecular weight, composition, crosslinking density, matrix geometry, and mechanical and rheological properties [10,11,43,44,45]. These matrices can also be modified with biofunctional chemical groups specific to the stem cell and differentiated cell types at a given developmental stage, thereby fine-tuning the scaffold for continued proliferation or differentiation [43]. The most commonly used polymers as precursor scaffolds are polyacrylamide (PAM), PEG, polylactic acid (PLA), poly(lactic-co-glycolic acid) (PLGA), polyhydroxyethyl methacrylate (PHEMA), and polyvinyl alcohol (PVA). In contrast, their synthetic derivatives were used in most of the stem cell-based applications [46,47]. PAM is an uncharged and bioinert substance that generally does not bind to the cell surface glycoproteins. PAM hydrogels can be tuned to varying stiffness and crosslinking capacity by incorporating different cell-adhesion peptides and biofunctional groups, thereby meeting user-defined requirements. However, due to the toxicity of PAM precursors, acrylamide and bisacrylamide, and the nature of polymerization, PAM cannot be used in 3D cell encapsulation and restricts its use only to 2D culture systems. PEG is another widely used synthetic hydrogel that has been used in most studies as an artificial scaffold. PEG is hydrophilic, bioinert, highly amenable to chemical modification, and nontoxic, making it a superior scaffold for 2D and 3D cell cultures (Figure 1A). Various polymerization chemistries, including photopolymerization, Michael addition, and enzymatic reactions, can be employed to produce PEG hydrogels with incorporated reactive groups [48,49,50,51].

**Figure 2:**
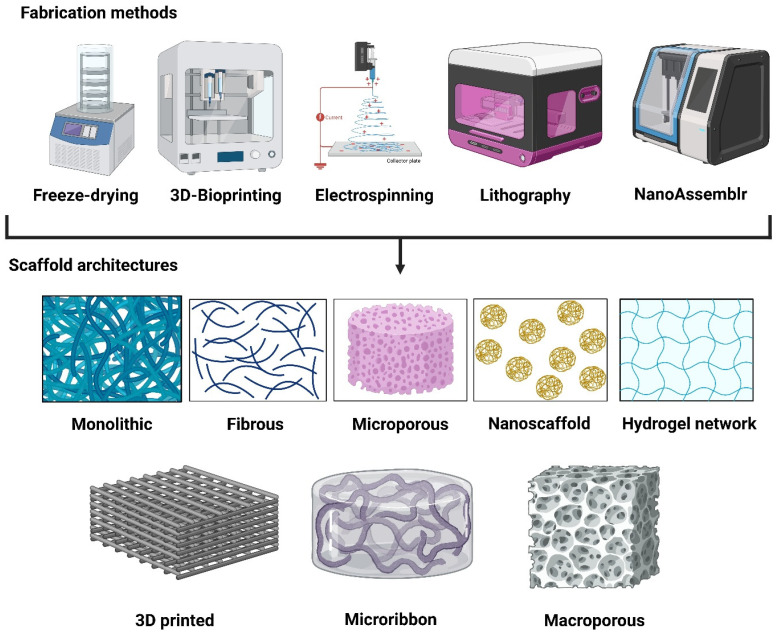
Fabrication methods of various synthetic engineered ECM scaffolds. Freeze-drying is used to fabricate microporous scaffolds; 3D bioprinting is used for microporous and 3D-printed scaffolds; fibrous scaffolds can be fabricated via electrospinning; lithography and NanoAssemblr are used for nanoscaffolds. Created in BioRender. Giri, S. (2026) https://BioRender.com/m6zijuf (accessed on 3 February 2026).

Synthetic ECM scaffolds are designed to recapitulate the biochemical, mechanical, and structural cues of native tissue microenvironments while offering extraordinary reproducibility and tunability. Different fabrication methods vary depending on the desired geometric architecture and tissue culture applications. The major fabrication approaches used to generate monolithic hydrogels, fibrous scaffolds, microribbons, and macroporous scaffolds are summarized in Figure 2. Monolithic hydrogels are continuous, non-porous networks typically formed through the bulk crosslinking of synthetic polymers (e.g., PEG, gelatin methacrylate, etc.) using UV or visible light photopolymerization, enzymatic reactions, or Michael-type additions, whereas rheometers and photoreactors are generally used for mechanical tuning [52]. To mimic the native ECM fibril architecture, nano- to micro-scale fibrous scaffolds are commonly fabricated through electrospinning using high-voltage power supplies, syringe pumps, and rotating collectors [53,54]. Microribbon scaffolds (μRB) consist of elongated hydrogel fragments produced by wet spinning or microfluidic extrusion followed by mechanical fragmentation and secondary crosslinking, enabling a high porosity and anisotropy (Figure 2). This type of scaffold is highly beneficial for bone and cartilage tissue or organoid culture [55,56]. In other types of scaffolds, microporous scaffolds are generated via porogen leaching, gas foaming, or cryogelation, using molds, freeze–thaw systems, and vacuum-drying equipment (Figure 2). In contrast, macroporous scaffolds with well-defined interconnected pores are often fabricated using 3D bioprinting or sacrificial templating with extrusion-based printers and temperature-controlled stages. Both such scaffolds provide 3D structural support, guide the self-assembly of stem cells to complex tissue-like structures, facilitate nutrient/waste exchange via pores, and improve organoid-based disease modeling and its applications in drug testing and regenerative medicine [57,58]. Other types of biomimetic scaffolds are nanoscaffolds that incorporate nanoscale features produced by self-assembly, phase separation, or nanolithography, using techniques such as peptide amphiphile assembly, spin coating, or atomic force microscopy-assisted patterning, as shown in Figure 2 [59,60]. Collectively, these fabrication approaches enable a precise control over scaffold mechanics, architecture, and biofunctionality to recapitulate tissue-specific ECM cues.

Scaffold architecture plays a critical role in regulating stem cell organization, fate specification, and lineage commitment by controlling 3D spatial confinement, ligand presentation, and mechanical cues within the cellular microenvironment. Certain architectural parameters such as porosity, pore size, fiber alignment, and dimensionality directly influence cell adhesion, migration, cytoskeletal organization, and other biochemical signaling pathways. Highly porous 3D-printed and electrospun scaffolds create interconnected channels that improve nutrient diffusion and facilitate cellular interactions. The porous scaffold has been proven effective for the efficient differentiation of hPSCs to pancreatic β-cell clusters and the myogenic differentiation of mesenchymal stem cells (MSCs) [58,61]. The size and shape of pores also play a critical role; for example, in bone regeneration biology, the smaller pores (~50–100 µm) support the cell attachment, while the larger ones (~200–400 µm) facilitate nutrient diffusion and angiogenesis [62]. The porous scaffolds with different pore geometries can also direct hMSCs to adopt distinct morphologies and differentiation outcomes. The octagonal architectures enhanced cell elongation and osteogenic differentiation more than hexagonal or square designs, illustrating that geometric arrangement can bias fate decisions [63]. Aligned fibrous scaffolds guide cell elongation and anisotropic organization, which efficiently differentiate cardiomyocytes from hiPSC-derived cardiac progenitor cells with improved functionality [64]. It also favors differentiation toward neural, tendon, or muscle lineages by activating the YAP/TAZ pathway and integrin-mediated signaling [65]. Similarly, electrospun and nanostructured scaffolds help hPSC aggregation, with an elevated adhesion to increased surface area contact, and promote efficient lineage commitment during differentiation [66,67]. Overall, scaffold structural parameters such as pore geometry, size, and fiber orientation act as biophysical signals that modulate stem cell organization and differentiation outcomes in tissue engineering contexts.

## 5. Synthetic ECM as a Superior Choice of Matrix for Human Pluripotent Stem Cell (hPSC) Culture

Human embryonic stem cells (hESCs) and induced pluripotent stem cells (hiPSCs) emerged as essential resources for modern disease modeling, drug discovery efforts, personalized medicine, regenerative medicine, and clinical applications [68,69]. hESCs/hiPSCs can proliferate indefinitely, are readily manipulated through genetic engineering, such as CRISPR gene editing, and retain the ability to differentiate into the three germ layers and, potentially, into any cell type in 2D or into specific organoids in 3D under a directed differentiation protocol. These characteristic attributes put hPSCs at the forefront of cutting-edge disease modeling, drug discovery, and clinical cell therapy [70,71]. Thriving hPSC culture depends on optimized culture protocols, chemically defined media, and matrix components in such applications. In the early days of hESC-based research, mouse embryonic fibroblasts (MEFs) served as feeder layers for hESCs. However, this co-culture system raised concerns about animal-origin contamination and spontaneous differentiation due to batch effects, which significantly affect culture efficiency. To mitigate this, the use of Matrigel as a basement membrane became popular, as it supports the proliferation and maintenance of pluripotency [72]. However, the undefined batch-to-batch variability and animal-derived xenogenic nature of Matrigel limit its clinical use in hPSC cultures, highlighting the advantages of fully synthetic matrix systems (Figure 1B).

In search of a Matrigel replacement for hPSC culture, a zwitterionic polymer, poly(2-(methacryloyloxy)ethyl dimethyl (3-sulfopropyl)ammonium hydroxide) (PMEDSAH), was first developed as a fully synthetic coating for long-term hPSC culture. It has been shown that PMEDSAH supports hPSC pluripotency and the gene expression network comparably to Matrigel, even after 20 passages [73,74]. In another study, a high-throughput screen of matrices identified a synthetic polymer poly(methyl vinyl ether-alt-maleic anhydride) (PMVE-alt-MA) that supports long-term attachment, the maintenance of pluripotency, and a normal karyotype, while reducing the spontaneous differentiation of hPSCs [75]. In addition to the matrix’s physical properties, the cell–matrix interaction properties were also investigated to develop an efficient surface coating material. It has been shown that hPSCs attach to Matrigel through integrin receptor subunits α5, α6, αv, β1, and β5 [76,77]. The fibronectin-derived three-amino-acid peptide Arg-Gly-Asp (RGD), which binds to both αvβ3 and αvβ5 integrins, provides a superior adhesion surface for hPSCs [78]. Improvements to the RGD scaffold were achieved through cyclization to cyclo(Arg-Gly-Asp-d-Phe-Lys) (cRGDfK), which is a more adhesive surface than standard laminin- or fibronectin-derived matrix peptides. In the same study, when cRGDfK was coupled with poly(acrylamide-co-propargyl acrylamide) (PAPA) brushes, it supported long-term hPSC cultures without affecting the pluripotency and karyotypic abnormalities [79]. In a subsequent study, cRGDfK was modified with 64 distinct PEG-thiol norbornene-based synthetic hydrogels and evaluated for hPSC cultures. Several candidates showed a better hPSC culture, maintenance, and preservation of the pluripotency [80].

Previous studies found that heparin sulfate proteoglycans can bind to basic fibroblast growth factor (bFGF) and protect it from proteolytic degradation, which promotes hPSC culture by maintaining the self-renewal ability and pluripotency [81,82,83]. Based on these findings, several efforts have been made to develop heparin sulfate-based synthetic hydrogels. One study developed a heparin-mimicking hydrogel, PAM6-co-PSS2, by copolymerizing poly(sodium 4-styrene sulfonate) (PSS) with PAM. This synthetic scaffold was able to support long-term hPSC culture in more than 20 passages with maintained pluripotency [84]. In 2010, a study found different proteoglycan-binding peptide sequences of GWQPPARARI, FHRRIKA, or GKKQRFRHRNRKG that can interact with cell surface glycosaminoglycans and support long-term hPSC self-renewal and pluripotency over three months [85]. In a later study, a PAM hydrogel was functionalized with a different vitronectin (VN)-derived peptide, GKKQRFRHRNRKG, and synthesized for elastic moduli (0.7, 3, and 10 kPa), and it has been shown that the rigid version (10 kPa) supports long-term hPSC cultures [86]. In addition, other VN-derived peptides were also conjugated with PAM/PEG hydrogels to produce an array of synthetic scaffolds. In a study, polyacrylic acid (PA) was used to coat the culture surface and was conjugated with a VN-derived peptide (Ac-KGGPQVTRGDVFTMP) and a bone sialoprotein-derived peptide (Ac-KGGNGEPRGDTYRAY) through EDC/NHS chemistry, which supported a long-term culture of multiple hPSC lines with chemically defined media [87]. Similarly, poly (OEGMA-co-HEMA) brushes were conjugated with VN peptides, which support the hiPSC culture over ten passages in a xeno-free and chemically defined media condition [88]. Another study used poly(vinyl alcohol-co-vinyl acetate-co-itaconic acid) (PVA) hydrogel grafted with a VN-derived peptide for hPSC cultures. It showed that it can support the hPSC culture for more than 20 passages in E8 media conditions [89]. In 2018, Sohi et al. used a chitosan film to immobilize VN-derived peptides and bFGF via an NHS-PEG-mal linker on its surface. They showed that it could support hiPSC self-renewal for an extended period in media containing FBS and bFGF [90]. In addition to these discoveries, a truncated version of human recombinant vitronectin (Vitronectin NC) was shown to be an excellent surface coating material for long-term hPSC cultures [91]. A study used carboxymethyl chitosan (CMC) on polydopamine-modified cell culture plates to immobilize the VN peptide via NHS/EDC chemistry. This coating material was proven to be supportive of hiPSC reprogramming and the long-term self-renewal (>20 passages) of multiple hPSC lines [92].

The hPSC culture system has evolved from 2D to 3D formats with advances in synthetic matrix technology. The 3D culture system promotes hPSC pluripotency and self-renewal by providing a better control over cell–cell and cell–matrix interactions, cellular morphology, and the culture microenvironment, which are crucial for hPSC growth [93,94,95,96]. In a pivotal study, the authors used an RGD-functionalized PEG hydrogel scaffold to culture hPSCs in a 3D format. They reported a 2.5-fold induction of pluripotency, reprogramming efficiency of human fibroblasts into hiPSCs, and homogeneous hiPSC colony generation [97]. In 3D scaffold preparation, electrospinning is a commonly used method. A 2014 study reported a layered gelatin nanofiber scaffold that supported an hPSC culture for more than 20 passages [98]. Later, this nanofiber was crosslinked with a microfiber layer composed of cellulose and polyglycolic acid, and the resulting scaffold supported hPSC survival for more than 2 months in mTeSR1 medium supplemented with Y27632 and methylcellulose [99]. In another study, PLGA and PMEDSAH were used to fabricate a nanofiber scaffold that supported hPSC adhesion and colony formation for two months without passaging [100]. Polystyrene electrospun fibers have also been used to develop a porous 3D scaffold that has been shown to support hiPSC culture for 10 passages [101]. The above examples of synthetic matrix hydrogels, other engineered scaffolds, and their applications in hPSC culture are summarized in Table 2.

## 6. Synthetic ECM as a Platform for Stem Cell Differentiation

With the advancement in our understanding of developmental biology, we are now more interested in stem cell-based organ-specific cell-type differentiation for in vitro disease modeling, drug screening, and regenerative medicine [69,102]. However, some challenges associated with stem cell culture, lineage-specific stem cell differentiation, the derivation of a homogeneous cell population, and providing a physiological microenvironment during culture and differentiation must be addressed before its clinical usage [103]. Previous evidence suggests that the surrounding microenvironment, especially the ECM composition and its physical and chemical characteristics, greatly influences stem cell differentiation towards lineage specificity [104,105]. The ill-defined composition of Matrigel is not the optimal choice for controlling microenvironmental cues during hPSC differentiation. To circumvent Matrigel-related challenges, researchers generated and tested synthetic ECMs and hydrogels to provide a tightly controlled microenvironmental scaffold for successful hPSC differentiation toward a homogeneous cell population. These synthetic ECMs and their application for lineage-specific hPSC differentiation will be discussed further.

### 6.1. Synthetic ECMs for Differentiation into Ectodermal Lineage

#### 6.1.1. Neuronal Lineage

The neural ECM has a pivotal role in neuronal cell proliferation, differentiation, maturation, and migration throughout brain development [106,107]. The neuronal ECM primarily consists of hyaluronic acid (HA), heparan sulfate proteoglycans, chondroitin sulfate proteoglycans (CSPGs), laminins, and reelin [108,109]. It has previously been shown that neuronal progenitor cells (NPCs) differentiate more efficiently in softer ECMs that mimic the native brain ECM composition [110,111,112]. HA-based hydrogel scaffolds have been used to photoencapsulate ventral midbrain-derived NPCs, accelerating the maturation of mechanical properties and ultimately differentiating into neurons [113]. The synthetic hydrogel PuraMatrix was used as an ECM microenvironment to differentiate hESCs into neurons and astrocytes [114]. Later, in another study, the authors functionalized PuraMatrix with a laminin-derived peptide sequence (-GGSDPGYIGSR-) and a bone marrow homing factor peptide sequence (-GGPFSSTKT-). This modified scaffold showed an increased neuronal cell proliferation, enhanced adhesion, and elevated survival [115]. The PEG-based hydrogel was also used as a micropatterned scaffold substrate to induce single neural rosette formation and to increase radial outgrowth with peripheral neuronal differentiation [116]. In another study, the authors used a PEG hydrogel formulation that contains maleimide-functionalized PEG with integrin-binding peptides and PEG-dithiol with MMP-degradable peptides, which support human astrocyte culture and maintenance in star-shaped morphologies [117]. PEG was also conjugated with a continuous gradient of an N-cadherin (NCAM)-derived peptide sequence (HAVDI), which was associated with increased survival, neurite extension, and neural differentiation in hiPSC-derived neural stem cells (hNSCs) [118]. PLGA-based fibers were used as a mesh to generate micropatterned embryoid bodies (EBs), thereby facilitating the guided self-organization of cortical plates in brain organoids [119]. Another synthetic substrate, a polyacrylamide (PAM) hydrogel with a stiffness of 0.7 kPa functionalized with a glycosaminoglycan (GAG)-binding peptide (CGKKQRFRHRNRKG), was shown to enhance neuronal differentiation from hESCs [120]. A poly-ε-caprolactone-based microsphere was used to encapsulate guggulsterone for sustained release during hiPSC differentiation toward a neuronal fate, resulting in increased TUJ1+/Olig2+ neuronal aggregates without affecting neurite length or branching [121]. A biocompatible group conjugated conductive polymer, Poly(3,4-ethylenedioxy thiophene) doped with poly(styrene sulfonic acid) (PEDOT: PSS), was also used to stimulate neuronal differentiation from hiPSC-derived neuronal progenitor cells electrically. PEDOT: PSS has been shown to support hNSC viability, enhance adhesion, promote neuron formation with longer neurites, and cause no apparent cytotoxicity [122,123]. Similarly, another conductive polymer, polypyrrole doped with anionic dodecylbenzene sulfonate (DBS), was used as a neuronal induction scaffold, and, upon electrical stimulation, hNSCs differentiated into neurons more efficiently, exhibiting longer neurite lengths, greater branching, and an upregulated expression of neurotrophic growth factors [124,125]. An electrospun PLGA membrane was fabricated with single-walled carbon nanotubes, and, upon electrical stimulation, hiPSCs were efficiently differentiated into NSCs, with an electrical conductivity controlling neuronal maturation [126]. The applications of the above-mentioned synthetic matrices for neuronal lineage differentiation are summarized in Table 3.

#### 6.1.2. Retinal Pigment Epithelial Cell Lineage

Retinal pigment epithelial cells arise from the developing anterior neuroectoderm, specifically from the anterior neural plate, during human development [131]. RPE cells have multimodal functions in the visual system, where they act as a physical barrier between the retina and choroidal blood vessels and play a crucial role in maintaining the photoreceptor (PR) conversion and storage of retinoids, the phagocytic removal of cellular debris, and other visual functions. RPE cell damage contributes to several degenerative eye diseases, including age-related macular degeneration (AMD). The advent of hESC/hiPSC-derived RPE cell differentiation in clinical settings provides an unprecedented opportunity for personalized curative therapies for AMD. In this context, the cGMP-compliant use of xeno-free materials, such as synthetic and defined ECMs, for RPE derivation is crucial. As in the literature, most studies have used Matrigel, laminin, poly-D-lysine, and fibronectin as ECM surfaces for hESC/hiPSC-derived RPE. The use of synthetic hydrogels and other synthetic ECMs holds promise for more efficient RPE derivation [132,133,134,135]. Scaffold-based approaches using synthetic or engineered ECM materials provide a prosthetic Bruch’s membrane that supports the formation of a polarized RPE monolayer, maintains cell–cell contacts, reduces immunogenic risk, and promotes long-term viability more effectively [136,137]. Additionally, synthetic ECM scaffolds can be designed with tunable mechanics and biodegradability to mimic native Bruch’s membrane permeability and to facilitate integration with host tissue, potentially improving transplantation outcomes and therapeutic efficacy in AMD [138].

For example, fibrin hydrogel was used to differentiate RPE from hiPSCs as a xeno-free, rapidly degradable synthetic ECM [139,140]. Another study tested four synthetic ECMs, like gelatin methacryloyl (GelMA), hyaluronic acid methacryloyl (HAMA), alginate, and fibrin hydrogels, for hESC-derived RPE adhesion and found that only fibrin hydrogel supports RPE adhesion [141]. In another study, human ARPE-19 cells were cultured on negatively charged poly (2-acrylamido-2-methyl propane sulfonic sodium) (PNaAMPS) and neutral poly(N, N-dimethylacrylamide) (PDMAAm) hydrogel surfaces and found that the usage of the PNaAMPS surface generates a very low level of reactive oxygen species (ROS) in the cultured RPE cells [142]. RGD-functionalized alginate hydrogel was also shown to elevate RPE cell differentiation in a 3D culture system [127]. PEG-grafted nanofiber surfaces have also supported RPE-cell attachment, proliferation, and maturation in vitro [128]. PEG/Gellan Gum (GG) hydrogel was also used as a biocompatible synthetic ECM surface for RPE cell attachment, survival, and proliferation [129]. In a recent study, several peptide fractions from vitronectin (PQVTRGDVFTMP) and the laminin β4 chain (PMQKMRGDVFSP) were grafted onto poly(vinyl alcohol-co-itaconic acid) (PAI) hydrogel and used as an ECM coating for hiPSC differentiation into RPE cells. The vitronectin-derived peptide fraction KVN2CK-grafted PAI hydrogel was found to support hiPSC differentiation into RPE cells, with an efficient proliferation and differentiation compared with Matrigel. These differentiated RPE cells were functional and were transplanted efficiently into a retinal degeneration rat model [130]. Table 3 summarizes the examples of synthetic ECMs that are promising candidates for RPE differentiation from hPSCs as 2D and 3D scaffolds, though they require further testing in future studies.

### 6.2. Synthetic ECMs for Differentiation into Endodermal Lineage

#### 6.2.1. Hepatic Lineages

Synthetic ECMs for hepatocyte differentiation from hiPSCs and mesenchymal stem cells (MSCs) and liver tissue regeneration are becoming increasingly popular in personalized medicine. Chemically defined media and xeno-free synthetic ECMs with tunable biochemical and mechanical cues continually improve the hepatic differentiation in 2D and 3D culture formats. In a study, a collagen-coated PLGA scaffold was used to support the hepatic differentiation of MSCs in 3D, resulting in a higher hepatic marker expression and more mature metabolic functions than 2D differentiation [143]. In another study, a PEG-based 3D scaffold was conjugated with fibronectin and collagen and efficiently induced hepatocyte differentiation from MSCs, compared with the 2D differentiation system [144]. Physical parameters, such as ECM stiffness, also play a crucial role in hepatocyte differentiation, in concert with ECM composition. A softer (400 Pa) 2D micropatterned heparin hydrogel was shown to support efficient hepatocyte differentiation and maturation from MSCs, compared with a stiffer (43 kPa) hydrogel [145]. Efficient hepatocyte differentiation from resident liver stem cells was achieved in a softer PA hydrogel (400 Pa) [146]. A collagen-coated PA hydrogel with moderate stiffness (20–140 kPa) has also been employed to successfully differentiate hepatocyte-like cells (HLCs) from ESCs, with increased albumin secretion and metabolic activity [147]. A 3D synthetic ECM scaffold made of PEG-diacrylate (PEGdA)-hyaluronic acid hydrogel conjugated with hydrolytically degradable GRGDS peptides with variable stiffness was used to encapsulate and differentiate primary hepatocyte progenitor cells, HepRG, towards mature hepatocytes. The synthetic scaffold, with a stiffness of 2.8–6.17 kPa that mimics native liver stiffness, enabled the more efficient differentiation and maturation of HepRG-derived hepatocytes [148].

The porosity and pore size of the 3D synthetic ECM scaffold also play a crucial biophysical cue for hepatocyte differentiation from hPSCs/MSCs. A synthetic 3D ECM nanofibrous scaffold composed of poly(L-lactic acid)-co-poly(ε-caprolactone) (PLACL)/collagen, with 89% porosity, efficiently transdifferentiated MSCs into hepatocytes compared with its counterparts when used as a standalone scaffold [149]. A biohybrid scaffold composed of hiPSC-derived HLCs and 3D melt–electrospun poly-ε-caprolactone (PCL) was used to mature hiPSC-derived HLCs into hepatocytes with key functional attributes, including albumin synthesis, cytochrome P450 activity, and glycogen storage [150]. A highly porous electrospun poly (L-lactic acid) (PLLA)/collagen nanofiber scaffold was also used to differentiate human bone marrow-derived MSCs (hBMSCs) into hepatocytes that expressed key hepatic lineage markers and functional attributes [151]. Similarly, an electrospun poly-ε-caprolactone/collagen/polyethersulfone nanofiber scaffold was more effective than a 2D culture system at transdifferentiating hBMSCs into functional hepatocytes [152]. In a previous study, a collagen I-infused bioplotting poly-l-lactic acid (PLLA) scaffold was used to differentiate hiPSCs into hepatocytes, which produced viable, polarized hepatic cells and also formed bile canaliculi-like structures; the functional maturation was lower than that of the hepatocytes differentiated on decellularized liver ECM [153]. In a recent study, a synthetic ‘click’ hydrogel was formulated using dendritic polyglycerol-bicyclononyne (dPG-BCN) and poly(N-isopropylacrylamide)-co-polyethylene glycol azide (pNIPAAm-co-PEG-N3), which was tunable and had biochemical properties. This hydrogel was conducive to long-term hiPSC proliferation, as well as to human liver organoid differentiation and lineage specification. RGD-conjugated versions of the same hydrogel promoted the differentiation of cholangiocytes from hiPSCs via enhanced TGF-β activation, whereas the RGD-free version promoted hepatocyte lineage differentiation [154]. In another recent study, the authors fabricated Biomimesys^®^, composed of collagen I/IV fibers, chemically modified HA, and Arginylglycylaspartic acid (RGDS) adhesion peptides. This hydrogel was shown to be effective for HLO differentiation within 28 days, with upregulated liver marker gene expression, increased cellular diversity, enhanced cytochrome P450 activities, and the production of apolipoprotein (a) [155]. Advances in the development of synthetic ECM for HLO and hepatocyte differentiation from hPSCs and hMSCs will enhance the adoption of xeno-free human liver models for hepatic disease research and drug discovery (Table 4).

#### 6.2.2. Intestinal Lineages

Human intestinal organoids (HIOs) differentiated from either hPSCs or adult Lgr5+ intestinal stem cells (ISCs) have emerged as an excellent tool for studying human intestinal development, disease modeling, in vitro drug testing, and regenerative medicine [170]. For consistent HIO generation and the use of xeno-free culture components in clinical applications, synthetic ECM with tunable stiffness, biochemical and mechanical cues, and biodegradability is gaining traction in HIO research. In 2017, a synthetic 4-arm PEG-maleimide-terminated hydrogel (PEG-4MAL) functionalized with an RGD peptide was used to differentiate HIOs from hPSCs. This fully defined hydrogel supported HIO differentiation from hiPSCs and maintained cell proliferation, apico-basal polarity, and structural complexity. The HIOs successfully engrafted onto the injured murine intestine and repaired the wound [41,42]. In a subsequent study, another synthetic PEG hydrogel was fabricated using an 8-arm vinyl sulfone-terminated PEG macromer (PEG-8VS) functionalized with a collagen I-mimicking adhesive peptide (GFOGER). It crosslinked with an MMP-degradable peptide to form primary human intestinal enteroids [156]. The enteroids retained proliferative capacity and apico-basal polarity and functionally responded to basolateral stimulation; they also expressed crypt cell- and Paneth cell-specific marker genes. Another synthetic PEG hydrogel was engineered through low-defect Thiol–Michael addition using an 8-arm PEG-VS hydrogel (2.5% *w*/*v*) containing an RGD peptide with an MMP-degradable crosslinker to grow patient-derived intestinal organoids [157]. Recently, a dynamic PEG-based hydrogel was developed using 8-arm PEG (8-PEG) as the backbone, with cytosine and vinyl sulfone covalently incorporated via Michael addition in a 50:50 ratio. This hydrogel, named Hybrid50, can reduce the stiffness of the 3D scaffold at a late stage of HIO development, thereby activating the YAP1 pathway and initiating symmetry breaking in HIOs [158]. In addition to different PEG hydrogels, a nonadhesive alginate hydrogel was used for hPSC-derived HIO differentiation and culture. This study first differentiated hPSCs toward the intestinal lineage on a 2D surface as spheroids. The spheroids were encapsulated in an alginate hydrogel to support continued differentiation and maturation toward the HIO fate. The functional and maturation profiles of these HIOs were comparable to those of HIOs differentiated in Matrigel as a 3D scaffold [169]. As a PEG-free 3D scaffold, a group recently developed a fully defined, tunable designer matrix, hyaluronan elastin-like protein (HELP), that supports the differentiation of HIOs from ISCs and the passaging of HIOs for a few generations [159]. The HELP hydrogel can be tuned for stiffness, the matrix stress–relaxation rate to support late-stage HIO development, and the matrix integrin–ligand concentration, providing layers of control for dynamic HIO development. As consolidated in Table 4, these synthetic ECMs can be used as 3D scaffolds and as a substitute for Matrigel in HIO differentiation.

#### 6.2.3. Pancreatic Lineage

To date, 3D pancreatic organoids are developed from hPSCs, pancreatic ductal cells, and embryonic pancreatic progenitors (PPs), primarily using Matrigel as an ECM scaffold [171,172,173]. However, as the ECM composition of native pancreatic islets differs in the endocrine and exocrine parts of the pancreas, the use of defined and tunable synthetic ECMs could enhance the differentiation of hPSCs/PPs towards functional and mature pancreatic organoids [174,175]. In an early study, an enzymatically crosslinked PEG hydrogel was shown to maintain and expand PPs but failed to support further pancreatic organoid maturation [172]. Later, another group designed a 3D scaffold composed of PEG-conjugated collagen I and encapsulated hESC-derived PPs. This scaffold supported long-term islet organoid culture and maintained organoid size and morphology [160]. An activin A grafted gelatin-PLGA nanofiber scaffold was also shown to differentiate hiPSCs to pancreatic cells efficiently [161]. Other synthetic scaffolds, such as PLLA/PVA and PCL/PVA nanofibrous scaffolds, were also tested and shown to support the enhanced differentiation of insulin-producing pancreatic cells from hiPSCs [162,163].

Alongside these synthetic scaffolds, alginate-based scaffolds were also used to differentiate hPSCs into pancreatic islets. In a recent study, the authors fabricated a 3D hydrogel scaffold, namely PVAMA-AlgMA-GelMA, using PVA, alginate, and gelatin conjugated with methacrylic anhydride (MA). The 3D scaffold was further modified by coating its surface with 75% activin A and BMP4. Subsequently, hiPSCs were encapsulated in this modified scaffold and treated with retinoic acid (RA)-loaded solid lipid nanoparticles, thereby differentiating hiPSC-derived definitive endoderms into pancreatic islets [164]. Alginate fibers were also used for the intraperitoneal transplantation of hiPSC-derived PP-spheroids in diabetic mice, resulting in the reversal of hyperglycemia [176]. Differentiation efficacy was also improved for hiPSC-derived pancreatic islets when hiPSCs were encapsulated in an alginate hydrogel [177]. Recently, a Na-Alginate-Chitosan hybrid capsule hydrogel was shown to facilitate the formation of hiPSC-differentiated, mature, and functional pancreatic organoids in an all-in-water microfluidic system. The resulting organoids consist of both pancreatic α- and β-cells, exhibit an elevated expression of pancreatic hormone-specific genes and proteins, and functionally secrete insulin in response to glucose [165]. Similarly, aqueous droplet-filled alginate calcium hydrogel fibers (ADHFs) were fabricated in a microfluidic system, and pancreatic endocrine progenitors were encapsulated in the ADHFs. The resulting pancreatic organoids exhibited a high viability and functional maturation, secreting insulin [166]. Apart from alginate-based hydrogel, recently, a novel hydrogel ‘Amikagel’ was formulated through ring-opening polymerization between amikacin hydrate and poly(ethylene glycol) diglycidyl ether (PEGDE). The resulting Amikagel facilitated spontaneous spheroid formation and the differentiation of mature pancreatic β-cells from hESCs. An increased expression of key pancreatic transcription factors was reported for PDX1 and NKX6.1, enriching the committed endocrine population. Upon further differentiation, Amikagel-derived spheroids yield β-like cells with higher INS gene and C-peptide levels and show glucose-responsive insulin secretion, indicating the improved functional maturation of β-cells in vitro [167]. The applications of the above-mentioned synthetic matrices are summarized in Table 4.

#### 6.2.4. Pulmonary Lineage

Synthetic ECMs are increasingly being adopted as alternatives to Matrigel for lung organoid culture, addressing limitations related to the undefined composition, variability, and lack of translational compatibility [178,179]. Among these, PEG-based hydrogels are the most extensively studied due to their excellent cytocompatibility, low immunogenicity, and modular chemistry that enables the independent control of stiffness, degradability, and ligand presentation [42]. Defined PEG systems, including PEG-4MAL and PEG-norbornene hydrogels functionalized with adhesive peptides and matrix metalloproteinase-sensitive crosslinkers, support human lung organoid formation comparably to Matrigel, promoting epithelial cyst growth, lumen formation, apicobasal polarization, and the expression of key lung lineage markers such as NKX2-1 and P63 [168,178]. The systematic tuning of matrix stiffness within these platforms has revealed that softer hydrogels approximating native lung mechanics preferentially enhance lung progenitor differentiation from hiPSCs, outperforming Matrigel-based cultures.

Beyond PEG, polysaccharide-based synthetic matrices such as alginate promote an increased airway epithelial diversity, yielding multiciliated, goblet, and basal cell populations organized in architectures that more closely resemble native airway tissue [169]. Additional synthetic polymers, including polyisocyanide (PIC), polyacrylamide, and polyvinyl alcohol hydrogels, offer further flexibility in fabrication and mechanical control [178]. Importantly, scaffold degradability has emerged as a critical determinant of lung organoid maturation. Non-degradable PEG-based microporous scaffolds restrict human lung organoid growth and maturation, largely maintaining cells in an immature NKX2.1^+^ progenitor state. In contrast, degradable polyester scaffolds such as PLG and PCL enable a robust organoid expansion and support the development of organized, pseudostratified airway epithelium. Following in vivo engraftment, organoids within these degradable matrices give rise to FOXJ1^+^ multiciliated cells and tissue architectures that closely resemble native airway structures [180]. Recent studies show that iPSC-derived alveolar organoids can be generated using synthetic hydrogel-based microwells and subsequently embedded within fully defined synthetic hydrogels. During both aggregation and embedding, these organoids actively secrete their own nascent extracellular matrix, creating a system that allows exogenous scaffold cues to be experimentally separated from cell-derived ECM signals [181]. Emerging synthetic, hybrid, and ECM-informed strategies continue to refine control over lung organoid development, bridging mechanistic insight with translational relevance.

A summary of synthetic scaffolds supporting hepatic, intestinal, pancreatic, and pulmonary differentiation from hPSCs is provided in Table 4.

### 6.3. Synthetic ECMs for Differentiation into Mesodermal Lineage

#### 6.3.1. Cardiac Lineages

In an early effort to advance hPSC-derived cardiac tissue differentiation protocols, thiolated hyaluronic acid (HA) was crosslinked with PEG-diacylate and used as a culture surface for cardiomyocyte (CM) maturation. The collagen I-coated synthetic HA-hydrogel was beneficial for pre-cardiac cell maturation, as it enhanced the maturation of muscle fibers by 60% and resulted in a threefold higher expression of cardiac-specific markers than a static PA hydrogel [182]. The direct encapsulation and differentiation of hiPSCs in a 3D scaffold composed of a gelatin methacryloyl (GelMA) hydrogel generated contractile CMs with well-defined, aligned sarcomeres and developmentally appropriate temporal maturation [183]. In a recent study, the surface of PLA microparticles was functionalized with poly(poly(ethylene glycol) methacrylate) and poly[N-(3-aminopropyl)methacrylamide] brushes, and this modified ECM surface enhanced hiMSC adhesion and hiPSC-derived cardiomyocyte adhesion and contractility [184]. Another study fabricated a PDMS-based elastomer stencil with a honeycomb pattern for hiPSC seeding and differentiation toward CMs. The results showed that 9 kPa of substrate stiffness induced embryoid-like aggregation, which led to an efficient CM differentiation [185]. The geometric pattern and topology of the culture scaffold play a critical role in efficient CM differentiation and maturation. In a study, hiPSCs were encapsulated in a PEG-fibrinogen scaffold in three geometrical patterns, like disk-shaped micro-islands, squares, and rectangles, and differentiated to CMs directly in the 3D scaffold. The resulting CMs from the rectangle 3D scaffold showed less tissue heterogeneity, advanced maturation features, such as myofibrillar alignment and Z-line formation, and higher anisotropic contractile properties [186]. Nanogrid culture arrays with nanogrooved topographies, with groove widths of 300–2000 nm were made of polyurethane acrylate (PUA) coated with RGD peptides and have also been successfully used to differentiate mature CMs from hiPSCs [187]. Another study used polydimethylsiloxane (PDMS) substrates with a sub-micrometer 3D topography and cylindrical geometric patterns for imprinting hiPSC-differentiated CMs and showed further accelerated differentiation and maturation [188]. A PDMS gel matrix coated with 1% Pluronic F127 was also used to fabricate tissue molds in which hiPSC-derived CMs were cultured, and it showed a high cell viability, greater 3D cell alignment, and enhanced maturation [189]. The applications of the above-mentioned synthetic matrices are summarized in Table 5.

Among all these synthetic ECMs, PEG-based hydrogels functionalized with MMP-degradable crosslinkers and integrin-binding RGD peptides are emerging as highly promising synthetic extracellular matrix platforms for future cardiac therapies. These materials combine FDA-approved biocompatibility with precisely tunable mechanical properties that can be matched to the native myocardial stiffness range (~3–15 kPa). In addition to supporting more reproducible cardioid formation, PEG hydrogels are compatible with minimally invasive, catheter-based delivery approaches for myocardial infarction treatment and can be manufactured under GMP-compliant conditions at clinically relevant scales. These features position PEG-based ECMs as strong candidates for future translational cardiac regenerative applications [204,205].

#### 6.3.2. Renal Lineages

Renal organoids, particularly kidney organoids differentiated from hPSCs, hold promise in regenerative medicine, as they can be used to study renal development and disease biology, support drug screening and development, and be engrafted in patients with chronic kidney disease. Although most kidney organoid generation protocols use different renal epithelial cells or primary human tubular epithelial cells as starting material, there is ongoing advancement in hPSC-differentiated kidney organoid development [206]. In parallel, the functional and physiological roles of scaffold ECMs are also being investigated in multiple studies. Renal organoids have successfully been generated in different natural, synthetic, and hybrid hydrogels, nanofiber scaffolds, and natural and decellularized ECM matrices, mostly from epithelial cells other than hPSCs [206]. The use of synthetic ECM scaffolds has been studied only recently; for example, one group used silk as a matrix for the differentiation of kidney organoids from hiPSCs [190]. The hiPSCs differentiated into renal epithelial cells that retained structural characteristics and supported the engraftment of renal organoids within the capsules of adult kidneys. Angiogenesis to the engraftment was elevated by using VEGF in the silk matrix, although the stromal cell differentiation was limited. In another study, the usage of a Thiol-ene crosslinked alginate hydrogel as an encapsulation scaffold for an hiPSC-differentiated kidney organoid resulted in an optimal expression of renal ECMs, like collagen 1A1, while maintaining all other aspects of kidney organoid physiology [191]. In a subsequent study, it was shown that a stiffer ECM (20 kPa) resulted in kidney organoids that lacked certain renal cell types, exhibited renal fibrosis signatures, and showed signs of epithelial-to-mesenchymal transition. However, when organoids were generated in a softer hydrogel (0.1 kPa), there was less deposition of ECM proteins, development of major renal segments, proximal tubule polarization, primary cilia formation, and functional maturation of the kidney organoid [192]. The results of this study confirm those of an earlier study, which showed that a softer, functionalized polyacrylamide hydrogel (1 kPa) enhanced the proliferation and maturation of kidney organoids compared with a stiffer PA hydrogel [193]. The applications of the above-mentioned synthetic matrices are consolidated in Table 5.

These studies indicate that carefully designed synthetic ECMs could enhance hPSC-differentiated kidney organoid maturation, engraftment, and functional repair, positing them as a promising platform for regenerative therapy (Table 5). The 3D ECM scaffolds were found to support the spatial organization of nephrons and promote the proper polarization of tubules and podocytes, making kidney organoids more mature with advanced renal function [207,208]. The properties like tunable stiffness, degradability, and biochemical cues in synthetic ECMs mimic the in vivo kidney microenvironment to promote vascularization, reduced fibrosis, and improved post-transplantation survival [209,210]. The kidney organoid embedded in the ECM scaffold can be engrafted under the renal capsule [211]. There, it can integrate with the host vasculature, show glomerular maturation, and exhibit limited urine filtration and solute handling, suggesting the potential for partial functional renal regeneration [212,213]. These synthetic ECMs can also act as a local drug or therapeutic agent delivery depot that modulates inflammation, fibrosis, and angiogenesis, further enhancing endogenous repair in chronic kidney injury models [214,215].

#### 6.3.3. Vascular Smooth Muscle Cell (vSMC) Lineage

The vSMCs form a layer of non-striated contractile mural cells, which provide mechanical strength to the endothelial layer of the blood vessels [216]. These vSMCs retain significant phenotypic plasticity, as they can stay proliferative and ECM-generating or as a force-generating contractile phenotype [217,218]. The stiffness and architecture of the synthetic ECM scaffold affect the fate of vascular smooth muscle cells (vSMCs) by modulating key cellular signaling pathways that sense mechanical forces through integrins and the cytoskeleton. The patterned and stiffer synthetic ECM scaffold enhances the formation of focal adhesions, and increased actomyosin tension activates RhoA/ROCK, FAK/Src, and the subsequent nuclear localization of YAP/TAZ [219,220]. This activation leads to the enhanced proliferation, migration, and activation of synthetic gene programs [221]. In contrast, softer or elastin-like structures diminish stress fiber formation, fostering a contractile and quiescent phenotype with an elevated expression of smooth muscle markers [222,223]. Additionally, nanotopography and the alignment of fibers further influence the cytoskeletal structure and nuclear shape, affecting mechanosensitive transcriptional responses and the transition between contractile and synthetic states [224,225].

hPSC-derived vSMCs are an essential tool for studying human vascular development and disease [226]. For the consistent differentiation of vSMCs from hPSCs, the use of synthetic ECM matrices may be beneficial. In one study, hiPSC-derived mural cells were differentiated in a 3D-GelMa scaffold with varying degrees of stiffness, functionalization, and crosslinking density. In a 3D-GelMa scaffold with a higher stiffness and crosslinking density, hiPSC-derived mural cells and vSMCs exhibited a contractile phenotype. They expressed key developmental markers similar to the primary human vSMCs and mural cells [194]. Another study used Poly(N-isopropylacrylamide) (PNIPAAm)-PEG and alginate hydrogels as a 3D scaffold for vSMC differentiation from hPSCs and found that the hydrogel-based culture system generated vSMCs with a better contractile phenotype and highly expressed genes related to vasculature development and angiogenesis compared to the 2D culture system [195]. A 3D macroporous nanofibrous PLLA scaffold has also been used to differentiate hiPSCs into vSMCs, and the study found that the differentiated vSMCs expressed SMC-specific marker genes and SMC phenotypes compared to the vSMCs differentiated from hiPSC with an all-trans retinoid acid (RA) induction batch [196,197]. These studies showed that the biochemical and mechanical properties of the culture matrix and scaffold are important developmental cues for vSMC development, which can be fine-tuned in synthetic ECM matrices rather than in Matrigel (Table 5).

#### 6.3.4. Osteoprogenitor Lineage

In recent years, numerous studies have sought to generate bone and musculoskeletal organoids from hPSCs and MSCs. The hiPSCs are an attractive source of starting material for personalized bone and cartilage regenerative therapy. For bone tissue differentiation and regeneration, different biocompatible and biodegradable polymeric scaffolds were investigated [227]. Fabricated bioactive hydrogel scaffolds are a great candidate for bone organoid generation, though they should (i) be biodegradable like natural ECM to promote cell adhesion, proliferation, and compatibility with osteogenic differentiation; (ii) be osteogenic, osteoconductive, and osteoinductive; (iii) be xeno-free, nonimmunogenic, and noncytotoxic; and (iv) have a porosity, stiffness and structural architecture compatible for osteogenesis [228,229,230]. Bone organoids generated using synthetic ECM scaffolds exhibit a stronger, more homogeneous mineralization and better regenerative potential than conventional matrices like Matrigel or inert porous scaffolds [227,231,232]. The Gelatin-Dextran hydrogel scaffold (G-PEG-Dx), GelMA-alendronate (GelMA-ALN), and DNA-Calcium phosphate hydrogels elevated ALP activity, RNX2/osteocalcin expression, and dense hydroxypartite-like deposition to enhance robust mineralization in bone organoids [233,234,235,236,237]. Tunable PEG-based synthetic hydrogels further enhance osteoblast differentiation and mineral deposition [238,239]. Upon engraftment, these organoids efficiently integrate with the host vasculature and form tubular-like bone structures more stable than rapidly degrading, mechanically weak traditional scaffolds.

In one study, iPSC-derived osteoprogenitor cells were encapsulated in a self-assembling synthetic peptide nanofiber hydrogel, PuraMatrix, and engrafted in a bone-injured rat model. The PuraMatrix was found to enhance vascularization and bone tissue regeneration at the injury site [198]. Two other studies supported this observation, in which PuraMatrix, as a hydrogel scaffold, promoted bone regeneration [199,200]. The iPSC-derived MSCs (hiPSC-MSCs) were also tested for osteogenic potential and compared with human dental pulp stem cells (hDPSCs) and bone marrow stem cells (hBMSCs). These cells were encapsulated in a calcium phosphate–alginate–fibrin hydrogel as an injectable scaffold. All stem cells showed a good proliferation and differentiation into the osteogenic lineage, although the osteogenic potential of hDPSCs was superior to that of the others [201]. Another study used HA- or collagen I-conjugated PEGdA and RGD-modified PEGdA hydrogels to differentiate hESCs into chondrogenic MSCs. The RGD-modified PEGdA hydrogel efficiently differentiated the neocartilage with basophilic extracellular matrix deposition compared to that of the other form of PEGdA hydrogel [202]. In a recent study, a 3D-bioprinted Ti6Al4V (3DTi) scaffold was used to rapidly differentiate osteocytes from hiPSCs via retinoic acid (RA)-induced osteogenesis, with a high reproducibility [203]. An osteogenesis-on-chip study fabricated a polymerized High Internal Phase Emulsion (polyHIPE) scaffold to differentiate woven bone organoids from hESC-derived mesenchymal progenitor cells (hES-MPs) by providing both biochemical and mechanical cues, such as flow and shear stress [240]. Because osteogenesis and bone regeneration are tightly coordinated by biochemical, mechanical, and structural signaling, the further development of tunable synthetic polymeric scaffolds will benefit future bone regeneration therapies (Table 5).

## 7. Synthetic ECM Properties Regulating Stem Cell Fate Specification

Individual ECM properties like stiffness, viscoelasticity, ligand presentation, degradability, and temporal dynamics systematically regulate stem cell fate decisions across diverse biological contexts. The integrated mechanotransduction pathways that translate biophysical cues into transcriptional programs bridge fundamental stem cell niche biology with rational material design principles.

**Figure 3:**
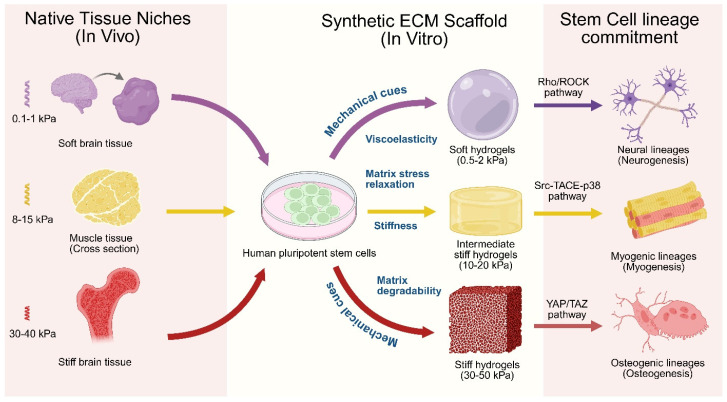
Mechanical properties of extracellular matrix and stem cell differentiation. Various mechanical properties of ECM act synchronously to influence the stem cell differentiation and lineage commitment. The activation of Rho/ROCK signaling pathway in soft hydrogel directs neuronal lineage commitment. Similarly, the activations of the Src–TACE–p38 pathway in medium stiffer hydrogels and the YAP/TAZ pathway in rigid hydrogels support the myogenic and osteogenic lineage commitment, respectively. Created in BioRender. Giri, S. (2026) https://BioRender.com/8ba0ezh (accessed on 9 February 2026).

**Stiffness:** Matrix stiffness serves as a primary biophysical determinant of human stem cell lineage specification, with substrates approximating tissue-specific elastic moduli (e.g., 0.1–1 kPa for brain, 8–17 kPa for muscle, 25–40 kPa for bone) [241], as illustrated in Figure 3. An integrin cytoskeletal-based mechanosensing mechanism regulates the fate and lineage specification [6,242]. The mechanotransduction cascade involves a physical linkage from ECM through integrin-based focal adhesions and cytoskeletal networks to the nucleus via LINC complexes [243]. The nuclear Lamin-A then systematically senses the tissue stiffness and directly couples the matrix mechanics to chromatin organization and gene expression [244]. The mechanotransduction pathways, like YAP/TAZ, FAK, and RhoA, regulated by tissue stiffness directly influence stem cell morphogenesis, differentiation, and organoid formation [221,241]. Synthetic ECM with an intermediate stiffness (2 kPa) was found to be conducive to efficient floor plate patterning in hiPSC-derived neural tube organoids compared to both softer (~0.7 kPa) and stiffer (~8 kPa) ECM scaffolds. Single cell transcriptional profiling and gene ontology (GO) analyses revealed that the tissue-driven ECM production, cytoskeleton organization, and planar cell polarity pathways were upregulated in the stretched neural tube organoid [245]. A PEG-based synthetic ECM with physiological stiffness (~1.3 kPa) was found to be optimal for intestinal organoid survival compared to a softer ECM (~0.2 kPa) [246]. In contrast, the softer ECM was found to be more efficiently conducive for crypt formation than the stiffer ones [247]. The YAP/Notch signaling was found to be responsive of ECM stiffness, modulating the crypt formation and differentiation of intestinal stem cells [248]. For liver organoids, PEG-based hydrogels efficiently differentiate hepatocytes in the liver organoids compared with Matrigel. The stiffer hydrogel (1.3 kPa) was found to enhance the hepatocyte functions in terms of albumin secretion and CYP450 activity, compared to that of softer hydrogels (0.3 kPa) [249]. Recent investigations revealed that stiff ECM (~40 kPa) promotes the osteogenic differentiation of MSCs by regulating energy metabolism through YAP-mediated mechanotransduction (Figure 3). YAP was found to function as a mechano-metabolic sensor that integrates mechanical cues with metabolic signaling pathways. It enhances glycolysis, oxidative phosphorylation, and mitochondrial fusion via increased mitofusin 1/2 expression while inhibiting dynamin-related protein 1 activity [250]. A follow-up study showed that the stiff matrices (25–50 kPa) favor osteogenesis in MSCs through the induction of RUNX2/ALP via 3D chromatin remodeling and merged TADs [251].

**Viscoelasticity:** Dynamic viscoelastic hydrogels that recapitulate the time-dependent mechanical cues of native tissues are proven to be superior scaffolds for organoid culture, surpassing the applicability of static ECM scaffolds [241,252,253]. The viscoelasticity depends on parameters like the loss modulus and rate of stress–relaxation. Hydrogels with enhanced stress relaxation promote MSC spreading, focal adhesion formation, osteogenic differentiation, and neural stem cell (NSC) astrocytic commitment through RhoA GTPase activity, actomyosin contractility, and focal adhesion signaling pathways (Figure 3) [254,255]. Viscoelastic matrices further enhance cellular plasticity by altering chromatin accessibility through nuclear lamina wrinkling, lamina-associated domain dissociation, and chromatin compaction [256]. Viscoelastic designs, such as alginate-PEG conjugates, high-molecular-weight (MW) alginate networks, and macroporous hydrogels enable stress–relaxation control through phototuning or rapid crosslink degradation [257,258]. Light-triggered PEG tethering to alginate spatially patterns relaxation rates (82–840 s half-times), guiding iPSC proliferation and morphology in 3D. Macroporous alginate (30% porosity) with BSA achieves an ultra-fast relaxation while retaining stability, enhancing nutrient diffusion for larger organoids. Such stress–relaxation promotes embryonic and branching morphogenesis via YAP/TAZ nuclear translocation and actomyosin remodeling [259]. For example, fast-relaxing PEG-alginate hydrogels (relaxation half-time ~1 min) or phototunable viscoelastic hydrogels accelerate intestinal organoid crypt–villus patterning and branching compared to elastic gels, mimicking embryonic gut ECMs [35,260]. DNA libraries with ultrahigh-MW polymers for tunable relaxation (1–100 s) were used to fabricate DNA-crosslinked matrix (DyNAtrix) hydrogel. DyNAtrix, being a dynamic viscoelastic hydrogel, supports the culture and development of human mesenchymal stromal cells, pluripotent stem cells, canine kidney cysts and human trophoblast organoids (GATA3+/GCM1+) with enhanced viability, proliferation, and morphogenesis [261]. During active cell cycle progression, the increased cellular volume activates stretch-activated ion channels, such as TRPV4 [262,263]. Rapid stress–relaxing hydrogels were found to reduce mechanical confinement and promote cell cycle progression through a TRPV4-PI3K/Akt-p27^Kip1^ signaling pathway [264]. In similar way, rapid stress relaxation promotes osteogenic differentiation from hMSCs through TRPV4 activation and the subsequent nuclear localization of RUNX2 [265].

**Ligand presentation:** Ligand presentation dynamics, such as ligand nanospacing, density, clustering, and temporal kinetics, systematically regulate stem cell fate decisions [266,267]. A critical nanospacing of approximately 60–70 nm determines the integrin clustering capacity and focal adhesion assembly, where larger spacings inhibits stable adhesion complex formation despite the high ligand density [266,268]. Photocontrolled supramolecular assemblies that dynamically inflate ligand nanospacing from 1.8 to 2.6 nm facilitate stem cell adhesion, mechanosensing, and differentiation both in vitro and in vivo [269]. Furthermore, slow peptide ligand (e.g., RGD peptide) dissociation kinetics enhance stem cell spreading and multi-directional migration compared to fast dissociation rates. Higher ligand clustering levels promote focal adhesion maturation and alkaline phosphatase expression in MSCs [270,271].

**Matrix degradability:** The matrix metalloproteinase (MMP) activity and tissue inhibitors of metalloproteinases (TIMPs) are primarily responsible for the regulation of ECM degradation to maintain tissue stiffness and viscoelasticity parameters. Matrix degradation regulates stem cell fate decisions by creating physical space for migration, releasing sequestered growth factors (e.g., VEGF, TGF-β, FGF-2), and modulating local mechanical properties within the stem cell niche [2,272]. MMP-degradable hydrogels enhance mesenchymal stem cell (MSC) migration and iPSC reprogramming efficiency. Highly degradable gels with an initial stiffness of 0.6 kPa, which is similar to embryonic tissue, yield the highest reprogramming outcomes through enhanced matrix malleability [40,262]. MMP activity enhances stress relaxation in both fibrillar collagen and synthetic matrices without altering bulk stiffness. The embedded cells sense such degradation-mediated mechanical alterations through modulated spreading and focal adhesion dynamics [273]. Engineered MMP-responsive hydrogels incorporating cell-mediated degradable crosslinkers enable dynamic ECM remodeling that recapitulates tissue development and regeneration processes. These synthetic ECMs are also compatible to controlled bioactive molecule release and angiogenic support, bridging stem cell niche biology with rational biomaterial design [274,275].

**Temporal dynamics:** The temporal dynamics of ECM cue presentation critically influence stem cell fate and lineage specification. The mechanosensing timescales, which can range from seconds (mechanosensor stretching, RhoA oscillations) to hours (gene expression) to weeks (permanent differentiation), systematically regulate stem cell fate decisions and lineage commitments [255,276]. Dynamic hydrogels with reversible stiffness revealed that neural stem cells exhibit a 24 h mechanosensitive window during which ECM stiffness profoundly impacts neurogenic commitment via substrate-dependent RhoA activation [255,277]. Skeletal muscle stem cells develop mechanical memory within the first three days of culture on stiff substrates, characterized by the RhoA-mediated loss of proliferative capacity that persists even after subsequent softening, demonstrating the temporal integration of mechanical memory [278]. Engineered hydrogels with dynamically tunable viscoelasticity through reversible crosslinks enable temporal control. Fast-dissociating crosslinks permit rapid force-induced network reorganization and enhanced mechanosensing compared to slow-dissociating crosslinks. Such binding kinetics critically determine stem cell spreading, differentiation, and chromatin accessibility across minute-to-day temporal regimes [279]. Synthetic ECMs incorporating phototunable, magnetoactive, or enzyme-responsive dynamic crosslinks now enable spatiotemporal stiffness pulsing that recapitulates developmental ECM remodeling, bridging stem cell and fate plasticity.

Modern engineered hydrogel design strategies leverage these principles with independently tunable stiffness, viscoelasticity, degradability, and bioactive ligand presentation. This enables a systematic high-throughput investigation of combinatorial ECM parameters that regulate stem cell fate, accelerated mesenchymal-to-epithelial transitions, and enhanced epigenetic remodeling during reprogramming [3,40]. These integrated ECM properties collectively define specialized microenvironments where heterogeneous cell populations, basement membrane components, and dynamic ECM remodeling converge to maintain stemness or direct differentiation through spatiotemporally orchestrated biophysical and biochemical signaling (Figure 3).

## 8. Recent Advances and Emerging Trends in Synthetic ECM Development

Advances in synthetic ECM engineering have fundamentally reshaped human stem cell and organoid culture by overcoming the variability and translational limitations of animal-derived materials such as Matrigel [35]. Fully defined synthetic matrices now enable reproducible control over mechanical properties, degradability, and biochemical signaling, allowing the in vitro reconstruction of key aspects of native tissue organization [41]. These developments, together with progress in stem cell biology and 3D bioprinting, have enabled the generation of organoids with improved structural fidelity, functional maturation, and clinical relevance. In recent times, a myriad of new approaches is being developed to advance synthetic ECM fabrication logics based on the knowledge of mechanical, physiochemical, architectural, and biochemical influence on human organoids and hPSC-derived cell cultures. The polymeric backbone of synthetic ECM is now generated through advanced polymerization chemistries like Michael additions, Thiol-ene chemistry, Diels–Alder (DA) reactions, inverse electron demand Diels–Alder (IEDDA) reactions, copper-catalyzed azide–alkyne cycloadditions (CuAAC), strain-promoted azide–alkyne cycloaddition (SPAAC) reactions, Sequential Click Reactions, and other bio-orthogonal chemistries [280,281]. Several key innate properties like the ECM stiffness, dynamic viscoelasticity, stress–relaxation through controlled biodegradation, and bioactive ligand presentation in a spatio-temporal fashion are now considered during synthetic ECM fabrication.

Emerging ECM strategies increasingly emphasize dynamic and cell-responsive signaling. Peptide amphiphile nanofibers functionalized with laminin-derived IKVAV motifs support the long-term maturation of human iPSC-derived neurons. Molecular mobility within the scaffold enhanced signal transduction beyond static ligand presentation [282]. Synthetic matrices also reduce organoid heterogeneity by enabling precise control over stiffness and composition, improving reproducibility in high-throughput and imaging-based applications. Consistent with this, neural organoids assembled on synthetic PEG hydrogels exhibit an enhanced cellular diversity, reduced batch variability, and functional immune responsiveness compared to Matrigel-based systems [283].

The integration of synthetic ECMs with advanced 3D bioprinting technologies has further advanced organoid engineering by enabling a spatially defined control over cell placement, matrix composition, and architecture [284]. Extrusion-, inkjet-, and light-based bioprinting approaches have been used to generate complex tissue geometries, including crypt–villus intestinal structures and cardiovascular-like models [285]. In particular, sacrificial writing into functional tissue (SWIFT) enables the incorporation of perfusable vascular channels into dense, cell-laden constructs, overcoming diffusion limitations and supporting long-term tissue viability and differentiation [286,287].

Artificial intelligence and machine learning (AI/ML) are increasingly reshaping synthetic extracellular matrix (ECM) design for organoid scaffolds by enabling data-driven optimization that replaces traditional trial-and-error approaches [288]. ML-guided models can predict and tune hydrogel composition, crosslinking chemistry, and mechanical properties, accelerating the development of ECM-mimetic bioinks with improved reproducibility, scalability, and printability for 3D bioprinting [289,290,291]. In parallel, AI tools streamline the analysis of high-dimensional biological data, including single-cell transcriptomics and multi-omics integration, providing deeper insight into organoid growth and differentiation [288]. The integration of machine learning with computer vision further enables real-time monitoring and adaptive control during bioprinting, improving fabrication robustness and consistency [292]. Emerging concepts such as ‘Organoid Intelligence’ extend these advances by coupling sensor-integrated brain organoids with ML-based training frameworks, highlighting future applications in biocomputing [37]. Despite significant progress, challenges remain related to data availability, model generalizability, experimental validation, and the integration of biological knowledge into computational pipelines [293].

## 9. Conclusions and Future Perspectives

Concerns about interpreting cell culture data generated using Matrigel were raised nearly three decades ago, yet its use as a 2D and 3D scaffold has continued to expand across cell biology and stem cell research [19]. The early reliance on Matrigel reflected the limited availability of alternative ECMs and the technical and resource barriers associated with developing synthetic matrices. Recent interdisciplinary advances, increased collaboration, and a growing demand for stem cell-based disease modeling have alleviated many of these challenges. Consequently, synthetic ECMs have emerged as robust alternatives, offering tunable biochemical and mechanical properties, controlled degradability, and defined biofunctionalization for reproducible hPSC culture, differentiation, and organoid generation. In parallel, improved embryoid body formation methods—such as bioreactors, rotary suspension culture, and microwell aggregation—now enable more homogeneous starting populations, enhancing lineage-specific organoid development [294,295].

PEG-based hydrogels have emerged as a leading synthetic platform due to their high cytocompatibility, resistance to nonspecific protein adsorption, and modular tunability. In particular, PEG-4MAL hydrogels, crosslinked with protease-degradable peptides and functionalized with adhesive motifs such as RGD, support the robust and reproducible generation of human intestinal organoids from pluripotent stem cells without Matrigel [41,42]. These matrices not only sustain organoid growth and differentiation but also enable minimally invasive delivery and engraftment in vivo, accelerating intestinal wound repair in murine models and highlighting their translational potential. Beyond PEG, fully synthetic PIC hydrogels with tunable charge and thermo-responsive behavior further expand the design space for defined organoid matrices [253]. Matrix mechanics play a central role in stem cell fate and morphogenesis. Soft PEG hydrogels with precisely tuned elastic moduli support the three-dimensional encapsulation of human pluripotent stem cells, preserving pluripotency and promoting uniform, lumenized aggregate formation, with intermediate stiffness favoring epiblast-like organization and a defined lower threshold required for cell viability [296]. In parallel, decellularized ECMs derived from native tissues retain tissue-specific biochemical and mechanical cues that are difficult to recapitulate synthetically. Pancreatic and kidney dECMs have been shown to enhance endocrine lineage assembly, vascularization, and the maturation of corresponding organoids, addressing persistent challenges in organoid size and functional integration [297,298]. Recent work further demonstrates that organoid formation can be driven by epithelial cell-secreted ECM, with endogenous laminin deposition establishing a de novo stem cell niche independent of exogenous matrix supplementation [299]. This finding provides a potential route toward eliminating xenogeneic materials and addressing regulatory barriers. Looking forward, the integration of synthetic ECM platforms with artificial intelligence and machine learning is poised to accelerate the optimization of bioink composition, printing parameters, and tissue architecture [300]. Key challenges remain, including scalable bioprinting, cost reduction, improved vascularization, and standardized protocols that balance synthetic control with biological complexity [301]. In this context, synthetic ECMs are best viewed as complementary tools that broaden the experimental landscape rather than as one-size-fits-all replacements for native or tissue-derived matrices. Apparently, no single engineered material can replicate the full biochemical richness and structural complexity found across all tissue niches. Instead, synthetic systems excel in contexts where researchers need tight control—such as isolating specific variables, probing mechanistic questions, or developing platforms suitable for scalable, translational manufacturing. Conversely, natural and decellularized ECMs may still be the preferred choice when robust, tissue-specific signaling or highly physiological remodeling dynamics are essential.

Recently, the U.S. Food and Drug Administration (FDA) provided the ‘Roadmap to Reducing Animal Testing in Preclinical Safety Studies’ (https://www.fda.gov/media/186092/download?attachment, accessed on 9 February 2026) and guidelines to embrace human-relevant disease models like organoids and organ-on-chip models under ‘New Approach Methodologies’ (NAMs) [302]. This brings unprecedented interest and demand for the development of organoid-based disease modeling platforms for fundamental research in disease biology and future drug development, across both public and private research entities. Alongside efforts to improve organoid culture reagents and recombinant growth factors/morphogens, significant efforts are underway to develop synthetic matrix scaffolds as alternatives to Matrigel. These efforts led to the establishment of new biotechnology startups, including Regenity, Advanced Biomatrix, Matricel GmbH, The Well Bioscience, Tissue Regenix, BioLamina AB, Luna-Gel™, and MIMETAS, as well as market leaders such as Thermo Scientific, Corning, and Sigma-Aldrich. Many of the synthetic matrices developed by these companies are described in this review. It is essential to continue developing bioengineered scaffolds through an open collaboration among stem cell biologists, materials scientists, clinicians, engineers, and biotechnology industry stakeholders. This will streamline production and commercial availability for end users in stem cell therapy, regenerative medicine, and the pharmaceutical industry.

## Figures and Tables

**Table 1:** Strengths and limitations of Matrigel, Natural ECMs, and Synthetic ECMs.

Criterion	Matrigel	Natural ECMs	Synthetic ECMs
Reproducibility	**Moderate:** Widely used ‘gold standard’ but has substantial lot-to-lot variability due to murine tumor origin and undefined composition.	**Low–moderate:** Composition depends on source tissue and decellularization protocol, leading to variability in protein content and mechanics.	**High:** Fully defined polymer networks with controlled chemistry and mechanics; minimal batch variability and highly consistent organoid formation.
Tunability (mechanics & ligands)	**Limited:** Stiffness and ligand content are difficult to tune independently; biochemical milieu cannot be precisely controlled.	**Moderate:** Some control via concentration, crosslinkers, or blending (e.g., collagen–gelatin), but mechanics and composition are coupled and harder to decouple.	**High:** Stiffness, viscoelasticity, degradability, and ligand density (RGD, laminin fragments, growth factor-binding domains) can be independently and dynamically tuned.
Biological complexity/bioactivity	**Very high:** Rich in laminin, collagen IV, entactin, and growth factors, supporting robust self-organization and differentiation across many organoid types.	**High:** Tissue-specific ECM composition preserves native cues (e.g., lung, liver, intestine), enhancing maturation and disease fidelity.	**Variable:** Intrinsically low bioactivity but can be engineered to present defined motifs; often needs supplementation to match Matrigel-level signaling.
Clinical translatability	**Poor:** Xenogeneic, tumor-derived, undefined; regulatory and safety concerns severely limit direct therapeutic use.	**Moderate:** Biologically relevant but still animal or donor derived, heterogeneous, and hard to standardize for GMP; immunogenicity is a concern.	**High potential:** Chemically defined, xeno-free, GMP-adaptable; better suited for regulatory approval and scalable manufacturing of transplantable organoids.
Scalability & automation	**Moderate:** Supports many protocols but temperature-sensitive, viscous, and difficult to handle in robotics and large-scale bioprocessing.	**Low–moderate:** Sourcing and batch processing of dECM are labor-intensive; variability complicates high-throughput and industrial workflows.	**High:** Liquid handling-friendly; compatible with microfluidics, droplet systems, and automated organoid production.
Cost	**Moderate:** Per unit but can be high at scale; waste due to handling constraints and batch testing adds hidden cost.	**Variable:** Collagen and fibrin relatively inexpensive; tissue-specific dECM can be costly in labor and QA/QC.	**Variable:** Some polymers are cheap at scale, but advanced functionalized or protein-engineered systems can be expensive to develop and synthesize.

**Table 2:** Synthetic bioengineered polymeric ECM materials and their application in hPSC culture.

Synthetic ECM Material	Cell Types & Application	Outcomes	Reference
PMEDSAH	hPSC culture, plate coating substrate	Supports long-term (>20 passages) culture with intact pluripotency	[73,74]
PMVE-alt-MA	hPSC culture, plate coating substrate	Supports long-term culture with normal pluripotency, karyotype, and reduced spontaneous differentiation	[75]
PAPA brushes attached with cRGDfk	hPSC culture, plate coating substrate	Supports long-term culture with normal pluripotency	[79]
PEG-thiol norbornene modified cRGDfk	hPSC culture, plate coating substrate	Better culture and pluripotency maintenance	[80]
PAM_6_-co-PSS2	hPSC culture, plate coating substrate	Supports long-term (>20 passages) culture with intact pluripotency	[84]
PAM functionalized with VN-derived peptide GKKQRFRHRNRKG	hPSC culture, plate coating substrate	Supports long-term hPSC culture with intact pluripotency and self-renewal capability	[86]
PA conjugated with VA peptide Ac-KGGPQVTRGDVFTMP or Ac-KGGNGEPRGDTYRAY	hPSC culture, plate coating substrate	Supports long-term hPSC culture with intact pluripotency and self-renewal capability	[87]
Poly (OEGMA-co-HEMA) brushes modified with VA peptides	hPSC culture, plate coating substrate	Supports long-term (>10 passages) hPSC culture in chemically defined media	[88]
PVA grafted with VA peptides	hPSC culture, plate coating substrate	Supports long term (>20 passages) hPSC culture in E8 media	[89]
VA peptides & bFGF immobilized on chitosan film	hPSC culture, plate coating substrate	Supports long-term hPSC culture in FBS/bFGF-containing media	[90]
Recombinant N-terminal truncated human vitronectin	hPSC culture, plate coating substrate	Supports long-term culture with normal pluripotency, karyotype, and reduced spontaneous differentiation	[91]
Immobilized VA peptides on CMC/polydopamine coating	hPSC culture, plate coating substrate	Supports long-term culture (>20 passages) with normal pluripotency, and reprogramming somatic cells to hiPSCs	[92]
RGD functionalized PEG hydrogels	hPSC culture in 3D format, scaffold	Induction of 2.5-fold pluripotency and homogeneous culture, higher reprogramming efficiency	[97]
Gelatin nanofiber	hPSC culture in 3D format, scaffold	Supports long-term culture (>20 passages) with normal pluripotency	[98]
Gelatin nanofiber crosslinked with cellulose & polyglycolic acid	hPSC culture in 3D format, scaffold	Supports long-term culture (>2 months) with mTeSR1 media supplemented with methylcellulose	[99]
PLGA/PMEDSAH nanofiber scaffold	hPSC culture in 3D format, scaffold	Supports long-term culture (>2 months), adherence, and colony formation	[100]
Polystyrene electrospun fiber	hPSC culture in 3D format, scaffold	Supports long-term culture (~10 passages)	[101]

**Table 3:** Synthetic bioengineered polymeric ECM materials and their application in ectodermal lineage-specific differentiation of hPSCs.

Synthetic ECM Material	Cell Types & Application	Outcomes	Reference
PuraMatrix	hESCs, neuronal differentiation	Efficient differentiation of hESCs to neurons and astrocytes	[114]
PuraMatrix functionalized with laminin peptide and bone marrow homing factor peptide	hESCs, neuronal differentiation	Increased neuronal cell proliferation, adhesion, and elevated survival	[115]
PEG micropatterned scaffold	hPSCs, neuronal differentiation	Induction of single neural rosette formation, increased radial outgrowth with peripheral neuronal differentiation	[116]
Maleimide functionalized PEG, PEG-dithiol tethered with MMP-degradable peptides	hPSCs, astrocyte	hPSC-derived human astrocyte culture and maintenance	[117]
PEG conjugated with NCAM-derived peptide HAVDI	hiPSCs, neuronal differentiation	Elevated survival, neurite extension, and neural differentiation from hiPSC-derived NSCs	[118]
PLGA fiber mesh	hiPSC, EB to brain organoid	Self-organization of the cortical plate in the brain organoid	[119]
PAM functionalized with GAG-binding peptide (CGKKQRFRHRNRKG)	hESCs, neuronal differentiation	Enhanced neuronal differentiation from hESCs	[120]
Poly-ε-caprolactone encapsulated guggulsterone	hiPSCs, neuronal differentiation	Elevated TUJ1+/Olig2+ neuronal aggregates with proper neurite length and branching	[121]
PEDOT: PSS conductive polymer	hiPSCs, neuronal differentiation	Higher neuronal progenitor cells’ viability, enhanced adhesion, efficient neuronal differentiation, longer neural dendrites	[122]
Polypyrrole: DBS conductive polymer	hiPSCs, neuronal differentiation	Efficient neuronal differentiation, longer neurites, more branching, and high expression of NGF	[124]
PLGA membrane fabricated with single-walled carbon nanotube	hiPSCs, neuronal differentiation	Upon electrical stimulation, efficient hiPSC differentiation to neuronal progenitor cells	[126]
RGD-alginate hydrogel	hiPSCs, retinal organoid development	Elevated retinal pigment epithelial cell differentiation from hiPSCs	[127]
PEG-grafted nanofibers	RPE cell maturation	Efficient maturation and proliferation of RPE cells	[128,129]
PEG/Gellan Gum hydrogel	RPE cell development	Efficient attachment, survival, and proliferation of RPE cells	[129]
Vitronectin-derived KVN2CK peptide grafted PAI hydrogel	hiPSCs, RPE differentiation	Efficient differentiation and proliferation of xeno-free RPE cells from hiPSCs	[130]

**Table 4:** Synthetic bioengineered polymeric ECM materials and their application in endodermal lineage-specific differentiation of hPSCs.

Synthetic ECM Material	Cell Types & Application	Outcomes	Reference
Collagen-coated PLGA scaffold	hMSCs, hepatic differentiation in 3D	Elevated hepatic marker expression and mature metabolic functions compared to the 2D format	[143]
Fibronectin & collagen conjugated PEG 3D scaffold	hMSCs, hepatic differentiation in 3D	Efficient hepatocyte differentiation	[144]
Collagen-coated PA hydrogel	hESCs, hepatic differentiation	Increased albumin secretion and metabolic activities	[147]
GRGDS peptide conjugated PEG-diacrylate (PEGdA)-hyaluronic acid hydrogel	Primary hepatocyte progenitor cells, hepatic differentiation	Efficient differentiation and maturation of HepRG-derived hepatocytes	[148]
(PLACL)/collagen nanofibrous 3D scaffold	hMSCs, hepatic differentiation	Efficient differentiation to hepatocytes	[149]
3D melt–electrospun poly-ε-caprolactone (PCL)	hiPSCs, hepatic differentiation	Efficient maturation of the hiPSC-derived HLCs to hepatocytes elevated albumin synthesis, cytochrome P450 activity, and glycogen storage	[150]
Electrospun (PLLA)/collagen nanofiber scaffold	hBMSCs, hepatic differentiation	Elevated hepatic marker gene expression and functional attributes	[151]
Electrospun poly-ε -caprolactone/collagen/polyethersulfone fabricated nanofiber scaffold	hBMSCs, hepatic differentiation	Efficient differentiation to hepatocytes in the 2D format	[152]
Bioplotted PLLA scaffold	hiPSCs, hepatic differentiation	Efficient differentiation of hiPSCs to hepatocytes with increased viability, polarization and structural complexity	[153]
(dPG-BCN)- (pNIPAAm-co-PEG-N3) hydrogel	hiPSCs, hepatic organoid differentiation	Efficient HLO differentiation	[154]
Biomimesys^®^	hiPSCs, hepatic organoid differentiation	Efficient HLO differentiation with elevated marker gene expression, cellular diversity, enhanced P450 activity, and apolipoprotein (a) production	[155]
PEG-4MAL functionalized with RGD peptide	hiPSCs, intestinal organoid differentiation	Support differentiation, maintain proliferation, apicobasal polarity, structural complexity, successful in vivo engraftment, and wound healing	[41,42]
PEG-8VS functionalized with GFOGER peptide crosslinked MMP-degradable peptide	hiPSCs, intestinal enteroid differentiation	Enteroids retained proliferative capacity, apicobasal polarity, express crypt and Paneth cell markers, responsive to basolateral stimulus	[156]
PEG-8VS functionalized with RGD peptide crosslinked MMP-degradable peptide	ISCs, patient-derived intestinal organoid	Efficient generation of HIO	[157]
Hybrid50 (8PEG-cytosine-vinyl sulfone)	ISCs, patient-derived intestinal organoid	Efficient generation of HIOs with elevated crypt budding and Paneth cell formation	[158]
Hyaluronan elastin-like protein (HELP)	ISCs, patient-derived intestinal organoid	Supports late stage HIO development	[159]
PEG-Collagen I	hESCs, PP to islet organoid differentiation	Supports long-term islet organoid culture with appropriate morphology and size	[160]
Activin A grafted gelatin-PLGA nanofiber scaffold	hiPSCs, pancreatic cell differentiation	Efficient differentiation of hiPSCs to insulin-producing pancreatic cells	[161]
PLLA/PVA & PCL/PVA nanofibrous scaffolds	hiPSCs, pancreatic cell differentiation	Efficient differentiation of hiPSCs to insulin-producing pancreatic cells	[162,163]
PVAMA-AlgMA-GelMA modified with activin A & BMP4	hiPSCs, pancreatic cell differentiation	Efficient differentiation of hiPSCs to insulin-producing pancreatic cells	[164]
Na-Alginate-Chitosan hybrid capsule hydrogel	hiPSCs, pancreatic cell differentiation	Mature and functional pancreatic organoid with α- and β-cells, exhibit glucose-stimulated insulin secretion	[165]
Aqueous droplet-filled alginate calcium hydrogel fibers (ADHFs)	PPs, pancreatic organoid differentiation	Pancreatic organoids exhibited high viability and functional maturation to secret insulin	[166]
Amikagel	hESC, pancreatic β-cell differentiation	Mature pancreatic β-cell with INS1 and c-peptide gene expression	[167]
PEG-NB hydrogel	hiPSC, NKX2.1+ lung progenitor (LP) differentiation	Soft hydrogels yield highest LP differentiation (54%), outperforming Matrigel	[168]
PEG-4MAL functional hydrogels	hPSC, lung organoid differentiation	Supports cyst formation, polarity, epithelial marker expression	[42]
Alginate hydrogel (2%)	hPSC, lung organoid differentiation	Produces greater epithelial diversity than Matrigel, including multiciliated and goblet cells	[169]

**Table 5:** Synthetic bioengineered polymeric ECM materials and their application in mesodermal lineage-specific differentiation of hPSCs.

Synthetic ECM Material	Cell Types & Application	Outcomes	Reference
GelMA scaffold	hiPSCs, cardiomyocyte (CM) differentiation	Contractile CMs with well-defined and aligned sarcomeres and temporal maturation	[183]
PLA microparticles functionalized with poly(PEG-MA) and poly[N-(3-aminopropyl)methacrylamide] brushes	hiPSCs, CM differentiation	Enhanced hiPSC derived CM adhesion and contractility	[184]
Honeycomb-patterned PDMS-based elastomer stencil	hiPSCs, CM differentiation	Efficient functional CM differentiation and maturation	[185]
Rectangular PEG-fibrinogen scaffold	hiPSCs, CM differentiation	More tissue homogeneity, advanced maturation features like myofibrillar alignment and Z-line formation, and enhanced anisotropic contractile properties	[186]
Nanogrids of PUA coated with RDG peptides	hiPSCs, CM differentiation	Efficient differentiation of mature CMs	[187]
PDMS sub µm 3D topography and cylindrical geometric patterns	hiPSCs, CM maturation	Accelerated differentiation and enhanced maturation of hiPSC-differentiated CMs	[188]
1% Pluronic F127-coated PDMS matrix	hiPSCs, CM maturation	High cell viability, greater 3D cell alignment, and enhanced maturation	[189]
Silk matrix w/wo VEGF	hiPSCs, kidney organoid differentiation	Efficient renal epithelial cell differentiation, supported renal organoid engraftment to adult kidney and angiogenesis	[190]
Thiol-ene crosslinked alginate	hiPSCs, kidney organoid differentiation	Efficient differentiation to mature kidney organoid with renal COL1A1 expression	[191]
Soft (<0.1 kPa) Na-Alginate	hiPSCs, kidney organoid differentiation	Development of major renal segments, proximal tubule polarization, primary cilia formation, and functional maturation of the kidney organoid	[192]
Functionalized soft (~1 kPa) PA hydrogel	hiPSCs, kidney organoid differentiation	Enhanced proliferation and maturation of kidney organoids	[193]
3D-GelMA scaffold of higher stiffness	hiPSCs, vSMC & mural cell differentiation	Mature vSMCs and mural cells with contractile phenotype, and expression of developmental markers	[194]
(PNIPAAm)-PEG and alginate hydrogel scaffold	hiPSCs, vSMC differentiation	Differentiated vSMCs with better contractile phenotype, vascular developmental gene expression, and angiogenesis in 3D format compared to 2D differentiation	[195]
Nanofibrous PLLA scaffold	hiPSCs, vSMC differentiation	Efficient vSMC differentiation with SMC phenotype and marker gene expression	[196,197]
PuraMatrix	hiPSCs, osteoprogenitor cell (OPC) maturation	Enhance vascularization and bone tissue regeneration of OPCs into the grafts	[198,199,200]
Calcium phosphate–alginate–fibrin hydrogel	hiPSC-MSCs/hDPSCs/hBMSCs, osteogenic differentiation	Superior osteogenic differentiation of hDPSCs compared to other stem cells	[201]
HA/Collagen I conjugated PEGdA, RGD-modified PEGdA hydrogel	hESCs, chondrogenic MSC differentiation	The MSCs efficiently differentiated to neocartilage with basophilic ECM deposition in the RGD-PEGdA scaffold	[202]
3D-bioprinted Ti6Al4V (3DTi) scaffold	hiPSCs, osteocyte differentiation	Enhanced differentiation to osteocytes with RA induction	[203]

## Data Availability

No new data were created or analyzed in this study.

## References

[1] Frantz C., Stewart K.M., Weaver V.M. (2010). The extracellular matrix at a glance. J. Cell Sci..

[2] Gattazzo F., Urciuolo A., Bonaldo P. (2014). Extracellular matrix: A dynamic microenvironment for stem cell niche. Biochim. Biophys. Acta.

[3] Watt F.M., Huck W.T. (2013). Role of the extracellular matrix in regulating stem cell fate. Nat. Rev. Mol. Cell Biol..

[4] Wang H., Luo X., Leighton J. (2015). Extracellular Matrix and Integrins in Embryonic Stem Cell Differentiation. Biochem. Insights.

[5] Wang L., Zheng F., Song R., Zhuang L., Yang M., Suo J., Li L. (2022). Integrins in the Regulation of Mesenchymal Stem Cell Differentiation by Mechanical Signals. Stem Cell Rev. Rep..

[6] Engler A.J., Sen S., Sweeney H.L., Discher D.E. (2006). Matrix elasticity directs stem cell lineage specification. Cell.

[7] Brielle S., Bavli D., Motzik A., Kan-Tor Y., Sun X., Kozulin C., Avni B., Ram O., Buxboim A. (2021). Delineating the heterogeneity of matrix-directed differentiation toward soft and stiff tissue lineages via single-cell profiling. Proc. Natl. Acad. Sci. USA.

[8] Xie Y.-H., Tang C.-Q., Huang Z.-Z., Zhou S.-C., Yang Y.-W., Yin Z., Heng B.C., Chen W.-S., Chen X., Shen W.-L. (2022). Extracellular Matrix Remodeling in Stem Cell Culture: A Potential Target for Regulating Stem Cell Function. Tissue Eng. Part B Rev..

[9] Lu P., Takai K., Weaver V.M., Werb Z. (2011). Extracellular matrix degradation and remodeling in development and disease. Cold Spring Harb. Perspect. Biol..

[10] Caliari S.R., Burdick J.A. (2016). A practical guide to hydrogels for cell culture. Nat. Methods.

[11] Lutolf M.P., Hubbell J.A. (2005). Synthetic biomaterials as instructive extracellular microenvironments for morphogenesis in tissue engineering. Nat. Biotechnol..

[12] Kleinman H.K., Martin G.R. (2005). Matrigel: Basement membrane matrix with biological activity. Semin. Cancer Biol..

[13] Kleinman H.K., McGarvey M.L., Hassell J.R., Star V.L., Cannon F.B., Laurie G.W., Martin G.R. (1986). Basement membrane complexes with biological activity. Biochemistry.

[14] Hughes C.S., Postovit L.M., Lajoie G.A. (2010). Matrigel: A complex protein mixture required for optimal growth of cell culture. Proteomics.

[15] Talbot N.C., Caperna T.J. (2015). Proteome array identification of bioactive soluble proteins/peptides in Matrigel: Relevance to stem cell responses. Cytotechnology.

[16] Timpl R., Rohde H., Robey P., Rennard S., Foidart J., Martin G. (1979). Laminin–a glycoprotein from basement membranes. J. Biol. Chem..

[17] Dirami G., Papadopoulos V., Kleinman H.K., Defreese D.C., Musto N.A., Dym M. (1995). Identification of transferrin and inhibin-like proteins in matrigel. Vitr. Cell. Dev. Biol. Anim..

[18] Hansen K.C., Kiemele L., Maller O., O’BRien J., Shankar A., Fornetti J., Schedin P. (2009). An in-solution ultrasonication-assisted digestion method for improved extracellular matrix proteome coverage. Mol. Cell. Proteom..

[19] Vukicevic S., Kleinman H.K., Luyten F.P., Roberts A.B., Roche N.S., Reddi A.H. (1992). Identification of multiple active growth factors in basement membrane matrigel suggests caution in interpretation of cellular activity related to extracellular matrix components. Exp. Cell Res..

[20] Elosegui-Artola A., Gupta A., Najibi A.J., Seo B.R., Garry R., Tringides C.M., de Lázaro I., Darnell M., Gu W., Zhou Q. (2023). Matrix viscoelasticity controls spatiotemporal tissue organization. Nat. Mater..

[21] Kane K.I.W., Moreno E.L., Lehr C.M., Hachi S., Dannert R., Sanctuary R., Wagner C., Fleming R.M.T., Baller J. (2018). Determination of the rheological properties of Matrigel for optimum seeding conditions in microfluidic cell cultures. AIP Adv..

[22] Alcaraz J., Xu R., Mori H., Nelson C.M., Mroue R., Spencer V.A., Brownfield D., Radisky D.C., Bustamante C., Bissell M.J. (2008). Laminin and biomimetic extracellular elasticity enhance functional differentiation in mammary epithelia. EMBO J..

[23] Reed J., Walczak W.J., Petzold O.N., Gimzewski J.K. (2009). In situ mechanical interferometry of matrigel films. Langmuir.

[24] Soofi S.S., Last J.A., Liliensiek S.J., Nealey P.F., Murphy C.J. (2009). The elastic modulus of Matrigel™ as determined by atomic force microscopy. J. Struct. Biol..

[25] Kohen N.T., Little L.E., Healy K.E. (2009). Characterization of Matrigel interfaces during defined human embryonic stem cell culture. Biointerphases.

[26] Borries M., Barooji Y.F., Yennek S., Grapin-Botton A., Berg-Sørensen K., Oddershede L.B. (2020). Quantification of Visco-Elastic Properties of a Matrigel for Organoid Development as a Function of Polymer Concentration. Front. Phys..

[27] Peterson N.C. (2008). From bench to cageside: Risk assessment for rodent pathogen contamination of cells and biologics. ILAR J..

[28] Liu H., Bockhorn J., Dalton R., Chang Y.-F., Qian D., Zitzow L.A., Clarke M.F., Greene G.L. (2011). Removal of lactate dehydrogenase-elevating virus from human-in-mouse breast tumor xenografts by cell-sorting. J. Virol. Methods.

[29] Riley V., Spackman D.H., Santisteban G.A., Dalldorf G., Hellstrom I., Hellstrom K.-E., Lance E.M., Rowson K.E.K., Mahy B.W.J., Alexander P. (1978). The LDH virus: An interfering biological contaminant. Science.

[30] Ammann C.G., Messer R.J., Peterson K.E., Hasenkrug K.J. (2009). Lactate dehydrogenase-elevating virus induces systemic lymphocyte activation via TLR7-dependent IFNalpha responses by plasmacytoid dendritic cells. PLoS ONE.

[31] Pineiro-Llanes J., da Silva L., Huang J., Cristofoletti R. (2024). Comparative Study of Basement-membrane Matrices for Human Stem Cell Maintenance and Intestinal Organoid Generation. J. Vis. Exp..

[32] Mayhew C.N., Singhania R. (2023). A review of protocols for brain organoids and applications for disease modeling. STAR Protoc..

[33] Fan X., Hou K., Liu G., Shi R., Wang W., Liang G. (2025). Strategies to overcome the limitations of current organoid technology—Engineered organoids. J. Tissue Eng..

[34] Lumibao J.C., Okhovat S.R., Peck K.L., Lin X., Lande K., Yomtoubian S., Ng I., Tiriac H., Lowy A.M., Zou J. (2024). The effect of extracellular matrix on the precision medicine utility of pancreatic cancer patient-derived organoids. JCI Insight.

[35] Gjorevski N., Sachs N., Manfrin A., Giger S., Bragina M.E., Ordóñez-Morán P., Clevers H., Lutolf M.P. (2016). Designer matrices for intestinal stem cell and organoid culture. Nature.

[36] Aisenbrey E.A., Murphy W.L. (2020). Synthetic alternatives to Matrigel. Nat. Rev. Mater..

[37] Smirnova L., Pantoja I.E.M., Hartung T. (2023). Organoid intelligence (OI)—The ultimate functionality of a brain microphysiological system. Altex.

[38] Kim S., Min S., Choi Y.S., Jo S.-H., Jung J.H., Han K., Kim J., An S., Ji Y.W., Kim Y.-G. (2022). Tissue extracellular matrix hydrogels as alternatives to Matrigel for culturing gastrointestinal organoids. Nat. Commun..

[39] Giobbe G.G., Crowley C., Luni C., Campinoti S., Khedr M., Kretzschmar K., De Santis M.M., Zambaiti E., Michielin F., Meran L. (2019). Extracellular matrix hydrogel derived from decellularized tissues enables endodermal organoid culture. Nat. Commun..

[40] Trappmann B., Gautrot J.E., Connelly J.T., Strange D.G., Li Y., Oyen M.L., Cohen Stuart M.A., Boehm H., Li B., Vogel V. (2012). Extracellular-matrix tethering regulates stem-cell fate. Nat. Mater..

[41] Cruz-Acuña R., Quirós M., Farkas E.A., Dedhia P.H., Huang S., Siuda D., García-Hernández V., Miller A.J., Spence J.R., Nusrat A. (2017). Synthetic hydrogels for human intestinal organoid generation and colonic wound repair. Nat. Cell Biol..

[42] Cruz-Acuña R., Quirós M., Huang S., Siuda D., Spence J.R., Nusrat A., García A.J. (2018). PEG-4MAL hydrogels for human organoid generation, culture, and in vivo delivery. Nat. Protoc..

[43] Li X., Sun Q., Li Q., Kawazoe N., Chen G. (2018). Functional Hydrogels With Tunable Structures and Properties for Tissue Engineering Applications. Front. Chem..

[44] Baker B.M., Chen C.S. (2012). Deconstructing the third dimension—How 3D culture microenvironments alter cellular cues. J. Cell Sci..

[45] Tibbitt M.W., Anseth K.S. (2009). Hydrogels as extracellular matrix mimics for 3D cell culture. Biotechnol. Bioeng..

[46] Unal A.Z., West J.L. (2020). Synthetic ECM: Bioactive Synthetic Hydrogels for 3D Tissue Engineering. Bioconjugate Chem..

[47] Madduma-Bandarage U.S.K., Madihally S.V. (2021). Synthetic hydrogels: Synthesis, novel trends, and applications. J. Appl. Polym. Sci..

[48] Nguyen K.T., West J.L. (2002). Photopolymerizable hydrogels for tissue engineering applications. Biomaterials.

[49] Schense J.C., Hubbell J.A. (1999). Cross-linking exogenous bifunctional peptides into fibrin gels with factor XIIIa. Bioconjugate Chem..

[50] Jivan F., Fabela N., Davis Z., Alge D.L. (2018). Orthogonal click reactions enable the synthesis of ECM-mimetic PEG hydrogels without multi-arm precursors. J. Mater. Chem. B.

[51] Zhang Z., Loebus A., de Vicente G., Ren F., Arafeh M., Ouyang Z., Lensen M.C. (2014). Synthesis of Poly(ethylene glycol)-based Hydrogels via Amine-Michael Type Addition with Tunable Stiffness and Postgelation Chemical Functionality. Chem. Mater..

[52] Zhang J., Yun S., Du Y., Zannettino A., Zhang H. (2020). Hydrogel-based preparation of cell aggregates for biomedical applications. Appl. Mater. Today.

[53] Ritzau-Reid K.I., Callens S.J.P., Xie R., Cihova M., Reumann D., Grigsby C.L., Prados-Martin L., Wang R., Moore A.C., Armstrong J.P.K. (2023). Microfibrous Scaffolds Guide Stem Cell Lumenogenesis and Brain Organoid Engineering. Adv. Mater..

[54] Jun I., Han H.-S., Edwards J.R., Jeon H. (2018). Electrospun Fibrous Scaffolds for Tissue Engineering: Viewpoints on Architecture and Fabrication. Int. J. Mol. Sci..

[55] Rogan H., Ilagan F., Tong X., Chu C.R., Yang F. (2020). Microribbon-hydrogel composite scaffold accelerates cartilage regeneration in vivo with enhanced mechanical properties using mixed stem cells and chondrocytes. Biomaterials.

[56] Gegg C., Yang F. (2020). Spatially patterned microribbon-based hydrogels induce zonally-organized cartilage regeneration by stem cells in 3D. Acta Biomater..

[57] Huang Y., Zhang X., Zhang W., Tang J., Liu J. (2025). Rational design matrix materials for organoid development and application in biomedicine. Regen. Biomater..

[58] Youngblood R.L., Sampson J.P., Lebioda K.R., Shea L.D. (2019). Microporous scaffolds support assembly and differentiation of pancreatic progenitors into β-cell clusters. Acta Biomater..

[59] Smith I.O., Liu X.H., Smith L.A., Ma P.X. (2009). Nanostructured polymer scaffolds for tissue engineering and regenerative medicine. Wiley Interdiscip. Nanomed. Nanobiotechnol..

[60] Han F., Meng Q., Xie E., Li K., Hu J., Chen Q., Li J., Han F. (2023). Engineered biomimetic micro/nano-materials for tissue regeneration. Front. Bioeng. Biotechnol..

[61] Pisani S., Marconi S., Mauri V., Rossetti B., Evangelista A., Bruni G., Benazzo M., Auricchio F., Conti B. (2025). Hybrid 3D-printed/electrospun scaffolds drive myogenic differentiation of mesenchymal stem cells (MSCs). Sci. Rep..

[62] Mukasheva F., Adilova L., Dyussenbinov A., Yernaimanova B., Abilev M., Akilbekova D. (2024). Optimizing scaffold pore size for tissue engineering: Insights across various tissue types. Front. Bioeng. Biotechnol..

[63] Guvendiren M., Fung S., Kohn J., De Maria C., Montemurro F., Vozzi G. (2017). The control of stem cell morphology and differentiation using three-dimensional printed scaffold architecture. MRS Commun..

[64] Ding M., Andersson H., Martinsson S., Sabirsh A., Jonebring A., Wang Q.-D., Plowright A.T., Drowley L. (2020). Aligned nanofiber scaffolds improve functionality of cardiomyocytes differentiated from human induced pluripotent stem cell-derived cardiac progenitor cells. Sci. Rep..

[65] Dalby M.J., Gadegaard N., Oreffo R.O.C. (2014). Harnessing nanotopography and integrin–matrix interactions to influence stem cell fate. Nat. Mater..

[66] Passos B.A.B.R., Battaglini M., Ciofani G. (2025). Nanostructured Biomaterial-Based Approaches to Support Induced Pluripotent Stem Cell Differentiation. Adv. NanoBiomed Res..

[67] Arrieta-Viana L.F., Mendez-Vega J., Torres-Lugo M. (2025). Synthetic thermoresponsive scaffolds for the expansion and differentiation of human pluripotent stem cells into cardiomyocytes. RSC Adv..

[68] Shi Y., Inoue H., Wu J.C., Yamanaka S. (2017). Induced pluripotent stem cell technology: A decade of progress. Nat. Rev. Drug Discov..

[69] Hoang D.M., Pham P.T., Bach T.Q., Ngo A.T.L., Nguyen Q.T., Phan T.T.K., Nguyen G.H., Le P.T.T., Hoang V.T., Forsyth N.R. (2022). Stem cell-based therapy for human diseases. Signal Transduct. Target. Ther..

[70] Avior Y., Sagi I., Benvenisty N. (2016). Pluripotent stem cells in disease modelling and drug discovery. Nat. Rev. Mol. Cell Biol..

[71] Singh V.K., Kalsan M., Kumar N., Saini A., Chandra R. (2015). Induced pluripotent stem cells: Applications in regenerative medicine, disease modeling, and drug discovery. Front. Cell Dev. Biol..

[72] Xu C., Inokuma M.S., Denham J., Golds K., Kundu P., Gold J.D., Carpenter M.K. (2001). Feeder-free growth of undifferentiated human embryonic stem cells. Nat. Biotechnol..

[73] Villa-Diaz L.G., Nandivada H., Ding J., Nogueira-de-Souza N.C., Krebsbach P.H., O’SHea K.S., Lahann J., Smith G.D. (2010). Synthetic polymer coatings for long-term growth of human embryonic stem cells. Nat. Biotechnol..

[74] Nandivada H., Villa-Diaz L.G., O’Shea K.S., Smith G.D., Krebsbach P.H., Lahann J. (2011). Fabrication of synthetic polymer coatings and their use in feeder-free culture of human embryonic stem cells. Nat. Protoc..

[75] Brafman D.A., Chang C.W., Fernandez A., Willert K., Varghese S., Chien S. (2010). Long-term human pluripotent stem cell self-renewal on synthetic polymer surfaces. Biomaterials.

[76] Meng Y., Eshghi S., Li Y.J., Schmidt R., Schaffer D.V., Healy K.E. (2010). Characterization of integrin engagement during defined human embryonic stem cell culture. FASEB J..

[77] Rowland T.J., Miller L.M., Blaschke A.J., Doss E.L., Bonham A.J., Hikita S.T., Johnson L.V., Clegg D.O. (2010). Roles of Integrins in Human Induced Pluripotent Stem Cell Growth on Matrigel and Vitronectin. Stem Cells Dev..

[78] Mondal G., Barui S., Chaudhuri A. (2013). The relationship between the cyclic-RGDfK ligand and αvβ3 integrin receptor. Biomaterials.

[79] Lambshead J.W., Meagher L., Goodwin J., Labonne T., Ng E., Elefanty A., Stanley E., O’bRien C.M., Laslett A.L. (2018). Long-Term Maintenance of Human Pluripotent Stem Cells on cRGDfK-Presenting Synthetic Surfaces. Sci. Rep..

[80] Nguyen E.H., Daly W.T., Le N.N.T., Farnoodian M., Belair D.G., Schwartz M.P., Lebakken C.S., Ananiev G.E., Saghiri M.A., Knudsen T.B. (2017). Versatile synthetic alternatives to Matrigel for vascular toxicity screening and stem cell expansion. Nat. Biomed. Eng..

[81] Furue M.K., Na J., Jackson J.P., Okamoto T., Jones M., Baker D., Hata R.-I., Moore H.D., Sato J.D., Andrews P.W. (2008). Heparin promotes the growth of human embryonic stem cells in a defined serum-free medium. Proc. Natl. Acad. Sci. USA.

[82] Spivak-Kroizman T., Lemmon M.A., Dikic I., Ladbury J.E., Pinchasi D., Huang J., Jaye M., Crumley G., Schlessinger J., Lax I. (1994). Heparin-induced oligomerization of FGF molecules is responsible for FGF receptor dimerization, activation, and cell proliferation. Cell.

[83] Vlodavsky I., Miao H.-Q., Medalion B., Danagher P., Ron D. (1996). Involvement of heparan sulfate and related molecules in sequestration and growth promoting activity of fibroblast growth factor. Cancer Metastasis Rev..

[84] Chang C.-W., Hwang Y., Brafman D., Hagan T., Phung C., Varghese S. (2013). Engineering cell–material interfaces for long-term expansion of human pluripotent stem cells. Biomaterials.

[85] Klim J.R., Li L., Wrighton P.J., Piekarczyk M.S., Kiessling L.L. (2010). A defined glycosaminoglycan-binding substratum for human pluripotent stem cells. Nat. Methods.

[86] Musah S., Morin S.A., Wrighton P.J., Zwick D.B., Jin S., Kiessling L.L. (2012). Glycosaminoglycan-Binding Hydrogels Enable Mechanical Control of Human Pluripotent Stem Cell Self-Renewal. ACS Nano.

[87] Melkoumian Z., Weber J.L., Weber D.M., Fadeev A.G., Zhou Y., Dolley-Sonneville P., Yang J., Qiu L., Priest C.A., Shogbon C. (2010). Synthetic peptide-acrylate surfaces for long-term self-renewal and cardiomyocyte differentiation of human embryonic stem cells. Nat. Biotechnol..

[88] Deng Y., Zhang X., Zhao X., Li Q., Ye Z., Li Z., Liu Y., Zhou Y., Ma H., Pan G. (2013). Long-term self-renewal of human pluripotent stem cells on peptide-decorated poly(OEGMA-co-HEMA) brushes under fully defined conditions. Acta Biomater..

[89] Higuchi A., Kao S.-H., Ling Q.-D., Chen Y.-M., Li H.-F., Alarfaj A.A., Munusamy M.A., Murugan K., Chang S.-C., Lee H.-C. (2015). Long-term xeno-free culture of human pluripotent stem cells on hydrogels with optimal elasticity. Sci. Rep..

[90] Sohi A.N., Naderi-Manesh H., Soleimani M., Yasaghi E.R., Manjili H.K., Tavaddod S., Nojehdehi S. (2018). Synergistic effect of co-immobilized FGF-2 and vitronectin-derived peptide on feeder-free expansion of induced pluripotent stem cells. Mater. Sci. Eng. C.

[91] Chen G., Gulbranson D.R., Hou Z., Bolin J.M., Ruotti V., Probasco M.D., Smuga-Otto K., Howden S.E., Diol N.R., Propson N.E. (2011). Chemically defined conditions for human iPSC derivation and culture. Nat. Methods.

[92] Zhou P., Wu F., Zhou T., Cai X., Zhang S., Zhang X., Li Q., Li Y., Zheng Y., Wang M. (2016). Simple and versatile synthetic polydopamine-based surface supports reprogramming of human somatic cells and long-term self-renewal of human pluripotent stem cells under defined conditions. Biomaterials.

[93] Gerecht S., Burdick J.A., Ferreira L.S., Townsend S.A., Langer R., Vunjak-Novakovic G. (2007). Hyaluronic acid hydrogel for controlled self-renewal and differentiation of human embryonic stem cells. Proc. Natl. Acad. Sci. USA.

[94] Ovadia E.M., Colby D.W., Kloxin A.M. (2018). Designing well-defined photopolymerized synthetic matrices for three-dimensional culture and differentiation of induced pluripotent stem cells. Biomater. Sci..

[95] Murphy W.L., McDevitt T.C., Engler A.J. (2014). Materials as stem cell regulators. Nat. Mater..

[96] Lei Y., Schaffer D.V. (2013). A fully defined and scalable 3D culture system for human pluripotent stem cell expansion and differentiation. Proc. Natl. Acad. Sci. USA.

[97] Caiazzo M., Okawa Y., Ranga A., Piersigilli A., Tabata Y., Lutolf M.P. (2016). Defined three-dimensional microenvironments boost induction of pluripotency. Nat. Mater..

[98] Liu L., Yoshioka M., Nakajima M., Ogasawara A., Liu J., Hasegawa K., Li S., Zou J., Nakatsuji N., Kamei K.-I. (2014). Nanofibrous gelatin substrates for long-term expansion of human pluripotent stem cells. Biomaterials.

[99] Liu L., Kamei K.-I., Yoshioka M., Nakajima M., Li J., Fujimoto N., Terada S., Tokunaga Y., Koyama Y., Sato H. (2017). Nano-on-micro fibrous extracellular matrices for scalable expansion of human ES/iPS cells. Biomaterials.

[100] Alamein M.A., Wolvetang E.J., Ovchinnikov D.A., Stephens S., Sanders K., Warnke P.H. (2015). Polymeric nanofibrous substrates stimulate pluripotent stem cells to form three-dimensional multilayered patty-like spheroids in feeder-free culture and maintain their pluripotency. J. Tissue Eng. Regen. Med..

[101] Leong M.F., Lu H.F., Lim T.C., Du C., Ma N.K., Wan A.C. (2016). Electrospun polystyrene scaffolds as a synthetic substrate for xeno-free expansion and differentiation of human induced pluripotent stem cells. Acta Biomater..

[102] Kimbrel E.A., Lanza R. (2020). Next-generation stem cells—Ushering in a new era of cell-based therapies. Nat. Rev. Drug Discov..

[103] Apostolou E., Blau H., Chien K., Lancaster M.A., Tata P.R., Trompouki E., Watt F.M., Zeng Y.A., Zernicka-Goetz M. (2023). Progress and challenges in stem cell biology. Nat. Cell Biol..

[104] Lutolf M.P., Gilbert P.M., Blau H.M. (2009). Designing materials to direct stem-cell fate. Nature.

[105] Zhang X., Zhang S., Wang T. (2022). How the mechanical microenvironment of stem cell growth affects their differentiation: A review. Stem Cell Res. Ther..

[106] Long K.R., Huttner W.B. (2019). How the extracellular matrix shapes neural development. Open Biol..

[107] Barros C.S., Franco S.J., Müller U. (2011). Extracellular matrix: Functions in the nervous system. Cold Spring Harb. Perspect. Biol..

[108] Zimmermann D.R., Dours-Zimmermann M.T. (2008). Extracellular matrix of the central nervous system: From neglect to challenge. Histochem. Cell Biol..

[109] Rutka J.T., Apodaca G., Stern R., Rosenblum M. (1988). The extracellular matrix of the central and peripheral nervous systems: Structure and function. J. Neurosurg..

[110] Saha K., Keung A.J., Irwin E.F., Li Y., Little L., Schaffer D.V., Healy K.E. (2008). Substrate Modulus Directs Neural Stem Cell Behavior. Biophys. J..

[111] Keung A.J., Asuri P., Kumar S., Schaffer D.V. (2012). Soft microenvironments promote the early neurogenic differentiation but not self-renewal of human pluripotent stem cells. Integr. Biol..

[112] Long K.R., Huttner W.B. (2022). The Role of the Extracellular Matrix in Neural Progenitor Cell Proliferation and Cortical Folding During Human Neocortex Development. Front. Cell. Neurosci..

[113] Seidlits S.K., Khaing Z.Z., Petersen R.R., Nickels J.D., Vanscoy J.E., Shear J.B., Schmidt C.E. (2010). The effects of hyaluronic acid hydrogels with tunable mechanical properties on neural progenitor cell differentiation. Biomaterials.

[114] Ylä-Outinen L., Joki T., Varjola M., Skottman H., Narkilahti S. (2014). Three-dimensional growth matrix for human embryonic stem cell-derived neuronal cells. J. Tissue Eng. Regen. Med..

[115] Liedmann A., Frech S., Morgan P.J., Rolfs A., Frech M.J. (2012). Differentiation of Human Neural Progenitor Cells in Functionalized Hydrogel Matrices. BioRes. Open Access.

[116] Knight G.T., Lundin B.F., Iyer N., Ashton L.M., Sethares W.A., Willett R.M., Ashton R.S. (2018). Engineering induction of singular neural rosette emergence within hPSC-derived tissues. eLife.

[117] Galarza S., Crosby A.J., Pak C., Peyton S.R. (2020). Control of Astrocyte Quiescence and Activation in a Synthetic Brain Hydrogel. Adv. Healthc. Mater..

[118] Lim H.J., Khan Z., Wilems T.S., Lu X., Perera T.H., Kurosu Y.E., Ravivarapu K.T., Mosley M.C., Callahan L.A.S. (2017). Human Induced Pluripotent Stem Cell Derived Neural Stem Cell Survival and Neural Differentiation on Polyethylene Glycol Dimethacrylate Hydrogels Containing a Continuous Concentration Gradient of *N*-Cadherin Derived Peptide His-Ala-Val-Asp-Ile. ACS Biomater. Sci. Eng..

[119] Lancaster M.A., Corsini N.S., Wolfinger S., Gustafson E.H., Phillips A.W., Burkard T.R., Otani T., Livesey F.J., Knoblich J.A. (2017). Guided self-organization and cortical plate formation in human brain organoids. Nat. Biotechnol..

[120] Musah S., Wrighton P.J., Zaltsman Y., Zhong X., Zorn S., Parlato M.B., Hsiao C., Palecek S.P., Chang Q., Murphy W.L. (2014). Substratum-induced differentiation of human pluripotent stem cells reveals the coactivator YAP is a potent regulator of neuronal specification. Proc. Natl. Acad. Sci. USA.

[121] Agbay A., De La Vega L., Nixon G., Willerth S.M. (2018). Guggulsterone-releasing microspheres direct the differentiation of human induced pluripotent stem cells into neural phenotypes. Biomed. Mater..

[122] Pires F., Ferreira Q., Rodrigues C.A., Morgado J., Ferreira F.C. (2015). Neural stem cell differentiation by electrical stimulation using a cross-linked PEDOT substrate: Expanding the use of biocompatible conjugated conductive polymers for neural tissue engineering. Biochim. Biophys. Acta (BBA)-Gen. Subj..

[123] Feig V.R., Santhanam S., McConnell K.W., Liu K., Azadian M., Brunel L.G., Huang Z., Tran H., George P.M., Bao Z. (2021). Conducting Polymer-Based Granular Hydrogels for Injectable 3D Cell Scaffolds. Adv. Mater. Technol..

[124] Stewart E., Kobayashi N.R., Higgins M.J., Quigley A.F., Jamali S., Moulton S.E., Kapsa R.M., Wallace G.G., Crook J.M. (2015). Electrical Stimulation Using Conductive Polymer Polypyrrole Promotes Differentiation of Human Neural Stem Cells: A Biocompatible Platform for Translational Neural Tissue Engineering. Tissue Eng. Part C Methods.

[125] Song S., Amores D., Chen C., McConnell K., Oh B., Poon A., George P.M. (2019). Controlling properties of human neural progenitor cells using 2D and 3D conductive polymer scaffolds. Sci. Rep..

[126] Landers J., Turner J.T., Heden G., Carlson A.L., Bennett N.K., Moghe P.V., Neimark A.V. (2014). Carbon nanotube composites as multifunctional substrates for in situ actuation of differentiation of human neural stem cells. Adv. Healthc. Mater..

[127] Hunt N.C., Hallam D., Karimi A., Mellough C.B., Chen J., Steel D.H., Lako M. (2017). 3D culture of human pluripotent stem cells in RGD-alginate hydrogel improves retinal tissue development. Acta Biomater..

[128] Tian Y., Zonca M.R., Imbrogno J., Unser A.M., Sfakis L., Temple S., Belfort G., Xie Y. (2017). Polarized, Cobblestone, Human Retinal Pigment Epithelial Cell Maturation on a Synthetic PEG Matrix. ACS Biomater. Sci. Eng..

[129] Kim H.S., Kim D., Jeong Y.W., Choi M.J., Lee G.W., Thangavelu M., Song J.E., Khang G. (2019). Engineering retinal pigment epithelial cells regeneration for transplantation in regenerative medicine using PEG/Gellan gum hydrogels. Int. J. Biol. Macromol..

[130] Liu J., Liu Q., Guo M., Jiang C., Chen J., Wang T., Sung T.-C., Chou S.-J., Chiou S.-H., Fan G. (2025). Differentiation of human induced pluripotent stem cells into retinal pigment epithelium cells during culture on peptide-grafted hydrogels. Regen. Biomater..

[131] Gupta S., Lytvynchuk L., Ardan T., Studenovska H., Faura G., Eide L., Znaor L., Erceg S., Stieger K., Motlik J. (2023). Retinal Pigment Epithelium Cell Development: Extrapolating Basic Biology to Stem Cell Research. Biomedicines.

[132] Lidgerwood G.E., Lim S.Y., Crombie D.E., Ali R., Gill K.P., Hernández D., Kie J., Conquest A., Waugh H.S., Wong R.C. (2016). Defined Medium Conditions for the Induction and Expansion of Human Pluripotent Stem Cell-Derived Retinal Pigment Epithelium. Stem Cell Rev. Rep..

[133] Osakada F., Jin Z.-B., Hirami Y., Ikeda H., Danjyo T., Watanabe K., Sasai Y., Takahashi M. (2009). In vitro differentiation of retinal cells from human pluripotent stem cells by small-molecule induction. J. Cell Sci..

[134] Idelson M., Alper R., Obolensky A., Ben-Shushan E., Hemo I., Yachimovich-Cohen N., Khaner H., Smith Y., Wiser O., Gropp M. (2009). Directed Differentiation of Human Embryonic Stem Cells into Functional Retinal Pigment Epithelium Cells. Cell Stem Cell.

[135] Zahabi A., Shahbazi E., Ahmadieh H., Hassani S.-N., Totonchi M., Taei A., Masoudi N., Ebrahimi M., Aghdami N., Seifinejad A. (2012). A New Efficient Protocol for Directed Differentiation of Retinal Pigmented Epithelial Cells from Normal and Retinal Disease Induced Pluripotent Stem Cells. Stem Cells Dev..

[136] Liu H., Huang S.S., Lingam G., Kai D., Su X., Liu Z. (2024). Advances in retinal pigment epithelial cell transplantation for retinal degenerative diseases. Stem Cell Res. Ther..

[137] Rizzolo L.J., Nasonkin I.O., Adelman R.A. (2022). Retinal Cell Transplantation, Biomaterials, and In Vitro Models for Developing Next-generation Therapies of Age-related Macular Degeneration. Stem Cells Transl. Med..

[138] Li Z., Hu Z., Gao Z. (2025). Advances in the Study of Age-Related Macular Degeneration Based on Cell or Cell-Biomaterial Scaffolds. Bioengineering.

[139] Gandhi J.K., Manzar Z., Bachman L.A., Andrews-Pfannkoch C., Knudsen T., Hill M., Schmidt H., Iezzi R., Pulido J.S., Marmorstein A.D. (2018). Fibrin hydrogels as a xenofree and rapidly degradable support for transplantation of retinal pigment epithelium monolayers. Acta Biomater..

[140] Gandhi J.K., Knudsen T., Hill M., Roy B., Bachman L., Pfannkoch-Andrews C., Schmidt K.N., Metko M.M., Ackerman M.J., Resch Z. (2019). Human Fibrinogen for Maintenance and Differentiation of Induced Pluripotent Stem Cells in Two Dimensions and Three Dimensions. Stem Cells Transl. Med..

[141] Wei Y., Alexandre U., Ma X. (2022). Hydrogels to Support Transplantation of Human Embryonic Stem Cell-Derived Retinal Pigment Epithelial Cells. Brain Sci..

[142] Chen Y.M., Liu Z.Q., Feng Z.H., Xu F., Liu J.K. (2014). Adhesive protein-free synthetic hydrogels for retinal pigment epithelium cell culture with low ROS level. J. Biomed. Mater. Res. Part A.

[143] Li J., Tao R., Wu W., Cao H., Xin J., Li J., Guo J., Jiang L., Gao C., Demetriou A.A. (2010). 3D PLGA scaffolds improve differentiation and function of bone marrow mesenchymal stem cell–derived hepatocytes. Stem Cells Dev..

[144] Wang Y., Lee J.-H., Shirahama H., Seo J., Glenn J.S., Cho N.-J. (2016). Extracellular Matrix Functionalization and Huh-7.5 Cell Coculture Promote the Hepatic Differentiation of Human Adipose-Derived Mesenchymal Stem Cells in a 3D ICC Hydrogel Scaffold. ACS Biomater. Sci. Eng..

[145] Hwang Y., Goh M., Kim M., Tae G. (2018). Injectable and detachable heparin-based hydrogel micropatches for hepatic differentiation of hADSCs and their liver targeted delivery. Biomaterials.

[146] Cozzolino A.M., Noce V., Battistelli C., Marchetti A., Grassi G., Cicchini C., Tripodi M., Amicone L. (2016). Modulating the Substrate Stiffness to Manipulate Differentiation of Resident Liver Stem Cells and to Improve the Differentiation State of Hepatocytes. Stem Cells Int..

[147] Mittal N., Tasnim F., Yue C., Qu Y., Phan D., Choudhury Y., Tan M.-H., Yu H. (2016). Substrate Stiffness Modulates the Maturation of Human Pluripotent Stem-Cell-Derived Hepatocytes. ACS Biomater. Sci. Eng..

[148] Lee H.-J., Son M.J., Ahn J., Oh S.J., Lee M., Kim A., Jeung Y.-J., Kim H.-G., Won M., Lim J.H. (2017). Elasticity-based development of functionally enhanced multicellular 3D liver encapsulated in hybrid hydrogel. Acta Biomater..

[149] Bishi D.K., Mathapati S., Venugopal J.R., Guhathakurta S., Cherian K.M., Ramakrishna S., Verma R.S. (2013). Trans-differentiation of human mesenchymal stem cells generates functional hepatospheres on poly(l-lactic acid)-co-poly(ε-caprolactone)/collagen nanofibrous scaffolds. J. Mater. Chem. B.

[150] Weber J., Linti C., Lörch C., Weber M., Andt M., Schlensak C., Wendel H.P., Doser M., Avci-Adali M. (2023). Combination of melt-electrospun poly-ε-caprolactone scaffolds and hepatocyte-like cells from footprint-free hiPSCs to create 3D biohybrid constructs for liver tissue engineering. Sci. Rep..

[151] Ghaedi M., Soleimani M., Shabani I., Duan Y., Lotfi A.S. (2012). Hepatic differentiation from human mesenchymal stem cells on a novel nanofiber scaffold. Cell. Mol. Biol. Lett..

[152] Kazemnejad S., Allameh A., Soleimani M., Gharehbaghian A., Mohammadi Y., Amirizadeh N., Jazayery M. (2009). Biochemical and molecular characterization of hepatocyte-like cells derived from human bone marrow mesenchymal stem cells on a novel three-dimensional biocompatible nanofibrous scaffold. J. Gastroenterol. Hepatol..

[153] Wang B., Jakus A.E., Baptista P.M., Soker S., Soto-Gutierrez A., Abecassis M.M., Shah R.N., Wertheim J.A. (2016). Functional Maturation of Induced Pluripotent Stem Cell Hepatocytes in Extracellular Matrix—A Comparative Analysis of Bioartificial Liver Microenvironments. Stem Cells Transl. Med..

[154] Wang L., Riediger L., Rao Q., Xu X., Nie Y., Zhou Y., Zhang J., Tang P., Wang W., Tacke F. (2025). Tunable Synthetic Hydrogel Modulates Hepatic Lineage Specification of Human Liver Organoid. Adv. Funct. Mater..

[155] Roudaut M., Caillaud A., Souguir Z., Bray L., Girardeau A., Rimbert A., Croyal M., Lambert G., Patitucci M., Delpouve G. (2024). Human induced pluripotent stem cells-derived liver organoids grown on a Biomimesys^®^ hyaluronic acid-based hydroscaffold as a new model for studying human lipoprotein metabolism. Bioeng. Transl. Med..

[156] Hernandez-Gordillo V., Kassis T., Lampejo A., Choi G., Gamboa M.E., Gnecco J.S., Brown A., Breault D.T., Carrier R., Griffith L.G. (2020). Fully synthetic matrices for in vitro culture of primary human intestinal enteroids and endometrial organoids. Biomaterials.

[157] Rezakhani S., Gjorevski N., Lutolf M.P. (2020). Low-Defect Thiol-Michael Addition Hydrogels as Matrigel Substitutes for Epithelial Organoid Derivation. Adv. Funct. Mater..

[158] Chrisnandy A., Blondel D., Rezakhani S., Broguiere N., Lutolf M.P. (2022). Synthetic dynamic hydrogels promote degradation-independent in vitro organogenesis. Nat. Mater..

[159] Hunt D.R., Klett K.C., Mascharak S., Wang H.Y., Gong D., Lou J., Li X., Cai P.C., Suhar R.A., Co J.Y. (2021). Engineered Matrices Enable the Culture of Human Patient-Derived Intestinal Organoids. Adv. Sci..

[160] Amer L.D., Holtzinger A., Keller G., Mahoney M.J., Bryant S.J. (2015). Enzymatically degradable poly(ethylene glycol) hydrogels for the 3D culture and release of human embryonic stem cell derived pancreatic precursor cell aggregates. Acta Biomater..

[161] Kuo Y.-C., Liu Y.-C., Rajesh R. (2017). Pancreatic differentiation of induced pluripotent stem cells in activin A-grafted gelatin-poly(lactide-co-glycolide) nanoparticle scaffolds with induction of LY294002 and retinoic acid. Mater. Sci. Eng. C Mater. Biol. Appl..

[162] Enderami S.E., Kehtari M., Abazari M.F., Ghoraeian P., Aleagha M.N., Soleimanifar F., Soleimani M., Mortazavi Y., Nadri S., Mostafavi H. (2018). Generation of insulin-producing cells from human induced pluripotent stem cells on PLLA/PVA nanofiber scaffold. Artif. Cells Nanomed. Biotechnol..

[163] Abazari M.F., Soleimanifar F., Aleagha M.N., Torabinejad S., Nasiri N., Khamisipour G., Mahabadi J.A., Mahboudi H., Enderami S.E., Saburi E. (2018). PCL/PVA nanofibrous scaffold improve insulin-producing cells generation from human induced pluripotent stem cells. Gene.

[164] Kuo Y.-C., Lee I.-H., Rajesh R. (2019). Self-assembled ternary poly(vinyl alcohol)-alginate-gelatin hydrogel with controlled-release nanoparticles for pancreatic differentiation of iPS cells. J. Taiwan Inst. Chem. Eng..

[165] Liu H., Wang Y., Wang H., Zhao M., Tao T., Zhang X., Qin J. (2020). A Droplet Microfluidic System to Fabricate Hybrid Capsules Enabling Stem Cell Organoid Engineering. Adv. Sci..

[166] Wang H., Liu H., Zhang X., Wang Y., Zhao M., Chen W., Qin J. (2021). One-Step Generation of Aqueous-Droplet-Filled Hydrogel Fibers as Organoid Carriers Using an All-in-Water Microfluidic System. ACS Appl. Mater. Interfaces.

[167] Candiello J., Grandhi T.S.P., Goh S.K., Vaidya V., Lemmon-Kishi M., Eliato K.R., Ros R., Kumta P.N., Rege K., Banerjee I. (2018). 3D heterogeneous islet organoid generation from human embryonic stem cells using a novel engineered hydrogel platform. Biomaterials.

[168] Tanneberger A.E., Blomberg R., Bilousova G., Ryan A.L., Magin C.M. (2025). Engineered hydrogel biomaterials facilitate lung progenitor cell differentiation from induced pluripotent stem cells. Am. J. Physiol. Cell. Mol. Physiol..

[169] Capeling M.M., Czerwinski M., Huang S., Tsai Y.-H., Wu A., Nagy M.S., Juliar B., Sundaram N., Song Y., Han W.M. (2019). Nonadhesive Alginate Hydrogels Support Growth of Pluripotent Stem Cell-Derived Intestinal Organoids. Stem Cell Rep..

[170] Taelman J., Diaz M., Guiu J. (2022). Human Intestinal Organoids: Promise and Challenge. Front. Cell Dev. Biol..

[171] Lee J., Sugiyama T., Liu Y., Wang J., Gu X., Lei J., Markmann J.F., Miyazaki S., Miyazaki J.-I., Szot G.L. (2013). Expansion and conversion of human pancreatic ductal cells into insulin-secreting endocrine cells. eLife.

[172] Greggio C., De Franceschi F., Figueiredo-Larsen M., Gobaa S., Ranga A., Semb H., Lutolf M., Grapin-Botton A. (2013). Artificial three-dimensional niches deconstruct pancreas development in vitro. Development.

[173] Huang L., Holtzinger A., Jagan I., BeGora M., Lohse I., Ngai N., Nostro C., Wang R., Muthuswamy L.B., Crawford H.C. (2015). Ductal pancreatic cancer modeling and drug screening using human pluripotent stem cell– and patient-derived tumor organoids. Nat. Med..

[174] Vigier S., Gagnon H., Bourgade K., Klarskov K., Fülöp T., Vermette P. (2017). Composition and organization of the pancreatic extracellular matrix by combined methods of immunohistochemistry, proteomics and scanning electron microscopy. Curr. Res. Transl. Med..

[175] Stendahl J.C., Kaufman D.B., Stupp S.I. (2009). Extracellular matrix in pancreatic islets: Relevance to scaffold design and transplantation. Cell Transplant..

[176] Fukuda S., Yabe S.G., Nishida J., Takeda F., Nashiro K., Okochi H. (2019). The intraperitoneal space is more favorable than the subcutaneous one for transplanting alginate fiber containing iPS-derived islet-like cells. Regen. Ther..

[177] Legøy T.A., Vethe H., Abadpour S., Strand B.L., Scholz H., Paulo J.A., Ræder H., Ghila L., Chera S. (2020). Encapsulation boosts islet-cell signature in differentiating human induced pluripotent stem cells via integrin signalling. Sci. Rep..

[178] Joo H., Min S., Cho S.-W. (2024). Advanced lung organoids for respiratory system and pulmonary disease modeling. J. Tissue Eng..

[179] Vazquez-Armendariz A.I., Tata P.R. (2023). Recent advances in lung organoid development and applications in disease modeling. J. Clin. Investig..

[180] Dye B.R., Youngblood R.L., Oakes R.S., Kasputis T., Clough D.W., Spence J.R., Shea L.D. (2020). Human lung organoids develop into adult airway-like structures directed by physico-chemical biomaterial properties. Biomaterials.

[181] Eiken M.K., Childs C.J., Brastrom L.K., Frum T., Plaster E.M., Ahmed D.W., Spencer R.C., Shachaf O., Pfeiffer S., Levine J.E. (2024). Nascent matrix deposition supports alveolar organoid formation from aggregates in synthetic hydrogels. Stem Cell Rep..

[182] Young J.L., Engler A.J. (2011). Hydrogels with time-dependent material properties enhance cardiomyocyte differentiation in vitro. Biomaterials.

[183] Kerscher P., Kaczmarek J.A., Head S.E., Ellis M.E., Seeto W.J., Kim J., Bhattacharya S., Suppiramaniam V., Lipke E.A. (2016). Direct Production of Human Cardiac Tissues by Pluripotent Stem Cell Encapsulation in Gelatin Methacryloyl. ACS Biomater. Sci. Eng..

[184] Alvarez-Paino M., Amer M.H., Nasir A., Crucitti V.C., Thorpe J., Burroughs L., Needham D., Denning C., Alexander M.R., Alexander C. (2019). Polymer Microparticles with Defined Surface Chemistry and Topography Mediate the Formation of Stem Cell Aggregates and Cardiomyocyte Function. ACS Appl. Mater. Interfaces.

[185] Wang B., Tu X., Wei J., Wang L., Chen Y. (2018). Substrate elasticity dependent colony formation and cardiac differentiation of human induced pluripotent stem cells. Biofabrication.

[186] Ellis M.E., Harris B.N., Hashemi M., Harvell B.J., Bush M.Z., Hicks E.E., Finklea F.B., Wang E.M., Nataraj R., Young N.P. (2022). Human Induced Pluripotent Stem Cell Encapsulation Geometry Impacts Three-Dimensional Developing Human Engineered Cardiac Tissue Functionality. Tissue Eng. Part A.

[187] Carson D., Hnilova M., Yang X., Nemeth C.L., Tsui J.H., Smith A.S., Jiao A., Regnier M., Murry C.E., Tamerler C. (2016). Nanotopography-Induced Structural Anisotropy and Sarcomere Development in Human Cardiomyocytes Derived from Induced Pluripotent Stem Cells. ACS Appl. Mater. Interfaces.

[188] Abadi P.P.S.S., Garbern J.C., Behzadi S., Hill M.J., Tresback J.S., Heydari T., Ejtehadi M.R., Ahmed N., Copley E., Aghaverdi H. (2018). Engineering of Mature Human Induced Pluripotent Stem Cell-Derived Cardiomyocytes Using Substrates with Multiscale Topography. Adv. Funct. Mater..

[189] Nakane T., Masumoto H., Tinney J.P., Yuan F., Kowalski W.J., Ye F., LeBlanc A.J., Sakata R., Yamashita J.K., Keller B.B. (2017). Impact of Cell Composition and Geometry on Human Induced Pluripotent Stem Cells-Derived Engineered Cardiac Tissue. Sci. Rep..

[190] Gupta A.K., Coburn J.M., Davis-Knowlton J., Kimmerling E., Kaplan D.L., Oxburgh L. (2019). Scaffolding kidney organoids on silk. J. Tissue Eng. Regen. Med..

[191] Geuens T., Ruiter F.A., Schumacher A., Morgan F.L., Rademakers T., Wiersma L.E., Berg C.W.v.D., Rabelink T.J., Baker M.B., LaPointe V.L. (2021). Thiol-ene cross-linked alginate hydrogel encapsulation modulates the extracellular matrix of kidney organoids by reducing abnormal type 1a1 collagen deposition. Biomaterials.

[192] Ruiter F.A.A., Morgan F.L.C., Roumans N., Schumacher A., Slaats G.G., Moroni L., LaPointe V.L.S., Baker M.B. (2022). Soft, Dynamic Hydrogel Confinement Improves Kidney Organoid Lumen Morphology and Reduces Epithelial–Mesenchymal Transition in Culture. Adv. Sci..

[193] Garreta E., Prado P., Tarantino C., Oria R., Fanlo L., Martí E., Zalvidea D., Trepat X., Roca-Cusachs P., Gavaldà-Navarro A. (2019). Fine tuning the extracellular environment accelerates the derivation of kidney organoids from human pluripotent stem cells. Nat. Mater..

[194] Meijer E.M., Giles R., van Dijk C.G.M., Maringanti R., Wissing T.B., Appels Y., Chrifi I., Crielaard H., Verhaar M.C., Smits A.I. (2023). Effect of Mechanical Stimuli on the Phenotypic Plasticity of Induced Pluripotent Stem-Cell-Derived Vascular Smooth Muscle Cells in a 3D Hydrogel. ACS Appl. Bio Mater..

[195] Liu Q., Liu Z., Gu H., Ge Y., Wu X., Zuo F., Du Q., Lei Y., Wang Z., Lin H. (2023). Comparative study of differentiating human pluripotent stem cells into vascular smooth muscle cells in hydrogel-based culture methods. Regen. Ther..

[196] Xie C., Hu J., Ma H., Zhang J., Chang L.-J., Chen Y.E., Ma P.X. (2011). Three-dimensional growth of iPS cell-derived smooth muscle cells on nanofibrous scaffolds. Biomaterials.

[197] Wang Y., Hu J., Jiao J., Liu Z., Zhou Z., Zhao C., Chang L.-J., Chen Y.E., Ma P.X., Yang B. (2014). Engineering vascular tissue with functional smooth muscle cells derived from human iPS cells and nanofibrous scaffolds. Biomaterials.

[198] Hayashi K., Ochiai-Shino H., Shiga T., Onodera S., Saito A., Shibahara T., Azuma T. (2016). Transplantation of human-induced pluripotent stem cells carried by self-assembling peptide nanofiber hydrogel improves bone regeneration in rat calvarial bone defects. BDJ Open.

[199] Nakahara H., Misawa H., Yoshida A., Hayashi T., Tanaka M., Furumatsu T., Tanaka N., Kobayashi N., Ozaki T. (2010). Bone repair using a hybrid scaffold of self-assembling peptide PuraMatrix and polyetheretherketone cage in rats. Cell Transplant..

[200] Kishimoto N., Momota Y., Mori R., Hashimoto Y., Imai K., Omasa T., Kotani J. (2008). Bone Regeneration Using Dedifferentiated Fat Cells with PuraMatrixTM. J. Oral Tissue Eng..

[201] Wang L., Zhang C., Li C., Weir M.D., Wang P., Reynolds M.A., Zhao L., Xu H.H. (2016). Injectable calcium phosphate with hydrogel fibers encapsulating induced pluripotent, dental pulp and bone marrow stem cells for bone repair. Mater. Sci. Eng. C Mater. Biol. Appl..

[202] Hwang N.S., Varghese S., Zhang Z., Elisseeff J. (2006). Chondrogenic Differentiation of Human Embryonic Stem Cell–Derived Cells in Arginine-Glycine-Aspartate–Modified Hydrogels. Tissue Eng..

[203] Yu L., Yang Y., Zhang B., Bai X., Fei Q., Zhang L. (2020). Rapid human-derived iPSC osteogenesis combined with three-dimensionally printed Ti6Al4V scaffolds for the repair of bone defects. J. Cell. Physiol..

[204] Song Y., Seitz M., Kowalczewski A., Mai N.Y., Jain E., Yang H., Ma Z. (2025). Mechanically and Chemically Defined PEG Hydrogels Improve Reproducibility in Human Cardioid Development. Adv. Healthc. Mater..

[205] Bhakuni S., Sharma H., Kim W., Kim D., Lee S., Park C., Oh J., Jeong H.E., Kim J. (2026). Micro and Nanoengineered Cardiac Spheroids and Organoids: Toward Translational Applications. Adv. Funct. Mater..

[206] Lacueva-Aparicio A., Lindoso R.S., Mihăilă S.M., Giménez I. (2022). Role of extracellular matrix components and structure in new renal models in vitro. Front. Physiol..

[207] van Sprang J.F., de Jong S.M.J., Dankers P.Y.W. (2022). Biomaterial-driven kidney organoid maturation. Curr. Opin. Biomed. Eng..

[208] Tabibzadeh N., Morizane R. (2024). Advancements in therapeutic development: Kidney organoids and organs on a chip. Kidney Int..

[209] Treacy N.J., Clerkin S., Davis J.L., Kennedy C., Miller A.F., Saiani A., Wychowaniec J.K., Brougham D.F., Crean J. (2023). Growth and differentiation of human induced pluripotent stem cell (hiPSC)-derived kidney organoids using fully synthetic peptide hydrogels. Bioact. Mater..

[210] Wang R., Sui Y., Liu Q., Xiong Y., Li S., Guo W., Xu Y., Zhang S. (2024). Recent advances in extracellular matrix manipulation for kidney organoid research. Front. Pharmacol..

[211] Freedman B.S., Dekel B. (2023). Engraftment of Kidney Organoids In Vivo. Curr. Transplant. Rep..

[212] Nakanoh H., Tsuji K., Fukushima K., Uchida N., Haraguchi S., Kitamura S., Wada J. (2025). Kidney Organoids: Current Advances and Applications. Life.

[213] Kalejaiye T.D., Barreto A.D., Musah S. (2022). Translating Organoids into Artificial Kidneys. Curr. Transplant. Rep..

[214] Jansen K., Schuurmans C.C., Jansen J., Masereeuw R., Vermonden T. (2017). Hydrogel-Based Cell Therapies for Kidney Regeneration: Current Trends in Biofabrication and In Vivo Repair. Curr. Pharm. Des..

[215] Cha S.-G., Rhim W.-K., Kim J.Y., Lee E.H., Lee S.Y., Park J.M., Lee J.E., Yoon H., Park C.G., Kim B.S. (2023). Kidney tissue regeneration using bioactive scaffolds incorporated with differentiating extracellular vesicles and intermediate mesoderm cells. Biomater. Res..

[216] Frismantiene A., Philippova M., Erne P., Resink T.J. (2018). Smooth muscle cell-driven vascular diseases and molecular mechanisms of VSMC plasticity. Cell. Signal..

[217] Majesky M.W. (2007). Developmental basis of vascular smooth muscle diversity. Arter. Thromb. Vasc. Biol..

[218] Cao G., Xuan X., Hu J., Zhang R., Jin H., Dong H. (2022). How vascular smooth muscle cell phenotype switching contributes to vascular disease. Cell Commun. Signal..

[219] Dieffenbach P.B., Haeger C.M., Coronata A.M.F., Choi K.M., Varelas X., Tschumperlin D.J., Fredenburgh L.E. (2017). Arterial stiffness induces remodeling phenotypes in pulmonary artery smooth muscle cells via YAP/TAZ-mediated repression of cyclooxygenase-2. Am. J. Physiol. Cell. Mol. Physiol..

[220] Cai X., Wang K.-C., Meng Z. (2021). Mechanoregulation of YAP and TAZ in Cellular Homeostasis and Disease Progression. Front. Cell Dev. Biol..

[221] Wang W., Lollis E.M., Bordeleau F., Reinhart-King C.A. (2019). Matrix stiffness regulates vascular integrity through focal adhesion kinase activity. FASEB J..

[222] Swiatlowska P., Sit B., Feng Z., Marhuenda E., Xanthis I., Zingaro S., Ward M., Zhou X., Xiao Q., Shanahan C. (2022). Pressure and stiffness sensing together regulate vascular smooth muscle cell phenotype switching. Sci. Adv..

[223] Shao Y., Li G., Huang S., Li Z., Qiao B., Chen D., Li Y., Liu H., Du J., Li P. (2020). Effects of Extracellular Matrix Softening on Vascular Smooth Muscle Cell Dysfunction. Cardiovasc. Toxicol..

[224] Chaterji S., Kim P., Choe S.H., Tsui J.H., Lam C.H., Ho D.S., Baker A.B., Kim D.-H. (2014). Synergistic effects of matrix nanotopography and stiffness on vascular smooth muscle cell function. Tissue Eng. Part A.

[225] Sazonova O.V., Isenberg B.C., Herrmann J., Lee K.L., Purwada A., Valentine A.D., Buczek-Thomas J.A., Wong J.Y., Nugent M.A. (2015). Extracellular matrix presentation modulates vascular smooth muscle cell mechanotransduction. Matrix Biol..

[226] Klein D. (2018). iPSCs-based generation of vascular cells: Reprogramming approaches and applications. Cell. Mol. Life Sci..

[227] Li C., Zhang Y., Du Y., Hou Z., Zhang Y., Cui W., Chen W. (2023). A Review of Advanced Biomaterials and Cells for the Production of Bone Organoid. Small Sci..

[228] Bai X., Gao M., Syed S., Zhuang J., Xu X., Zhang X.-Q. (2018). Bioactive hydrogels for bone regeneration. Bioact. Mater..

[229] Yue S., He H., Li B., Hou T. (2020). Hydrogel as a Biomaterial for Bone Tissue Engineering: A Review. Nanomaterials.

[230] Kim H.D., Amirthalingam S., Kim S.L., Lee S.S., Rangasamy J., Hwang N.S. (2017). Biomimetic Materials and Fabrication Approaches for Bone Tissue Engineering. Adv. Healthc. Mater..

[231] Han Y., Liu J., Hu C., Wang Y., He C. (2025). Advances in hydrogel systems for bone regeneration: Trends, innovations, and prospects. J. Mater. Chem. B.

[232] Hong Y., Li R., Sheng S., Zhou F., Bai L., Su J. (2025). Bone organoid construction and evolution. J. Orthop. Transl..

[233] Liu L., Li X., Shi X., Wang Y. (2018). Injectable alendronate-functionalized GelMA hydrogels for mineralization and osteogenesis. RSC Adv..

[234] Zhou B., Jiang X., Zhou X., Tan W., Luo H., Lei S., Yang Y. (2023). GelMA-based bioactive hydrogel scaffolds with multiple bone defect repair functions: Therapeutic strategies and recent advances. Biomater. Res..

[235] Re F., Sartore L., Borsani E., Ferroni M., Baratto C., Mahajneh A., Smith A., Dey K., Almici C., Guizzi P. (2021). Mineralization of 3D Osteogenic Model Based on Gelatin-Dextran Hybrid Hydrogel Scaffold Bioengineered with Mesenchymal Stromal Cells: A Multiparametric Evaluation. Materials.

[236] Gai T., Zhang H., Hu Y., Li R., Wang J., Chen X., Wang J., Chen Z., Jing Y., Wang C. (2025). Sequential construction of vascularized and mineralized bone organoids using engineered ECM-DNA-CPO-based bionic matrix for efficient bone regeneration. Bioact. Mater..

[237] Wu X., Hu Y., Sheng S., Yang H., Li Z., Han Q., Zhang Q., Su J. (2025). DNA-based hydrogels for bone regeneration: A promising tool for bone organoids. Mater. Today Bio.

[238] Chatterjee K., Lin-Gibson S., Wallace W.E., Parekh S.H., Lee Y.J., Cicerone M.T., Young M.F., Simon C.G. (2010). The effect of 3D hydrogel scaffold modulus on osteoblast differentiation and mineralization revealed by combinatorial screening. Biomaterials.

[239] Si J., Ishikawa S., Nepal S., Okada H., Chung U.-I., Sakai T., Hojo H. (2025). Osteogenic differentiation capabilities of multiarm PEG hydrogels: Involvement of gel–gel-phase separation in cell differentiation. Polym. J..

[240] Bahmaee H., Owen R., Boyle L., Perrault C.M., Garcia-Granada A.A., Reilly G.C., Claeyssens F. (2020). Design and Evaluation of an Osteogenesis-on-a-Chip Microfluidic Device Incorporating 3D Cell Culture. Front. Bioeng. Biotechnol..

[241] Lai W., Geliang H., Bin X., Wang W. (2025). Effects of hydrogel stiffness and viscoelasticity on organoid culture: A comprehensive review. Mol. Med..

[242] Discher D.E., Mooney D.J., Zandstra P.W. (2009). Growth factors, matrices, and forces combine and control stem cells. Science.

[243] Sun Z., Guo S.S., Fässler R. (2016). Integrin-mediated mechanotransduction. J. Cell Biol..

[244] Swift J., Ivanovska I.L., Buxboim A., Harada T., Dingal P.C.D.P., Pinter J., Pajerowski J.D., Spinler K.R., Shin J.-W., Tewari M. (2013). Nuclear lamin-a scales with tissue stiffness and enhances matrix-directed differentiation. Science.

[245] Abdel Fattah A.R., Daza B., Rustandi G., Berrocal-Rubio M.Á., Gorissen B., Poovathingal S., Davie K., Barrasa-Fano J., Cóndor M., Cao X. (2021). Actuation enhances patterning in human neural tube organoids. Nat. Commun..

[246] Hushka E.A., Yavitt F.M., Brown T.E., Dempsey P.J., Anseth K.S. (2020). Relaxation of Extracellular Matrix Forces Directs Crypt Formation and Architecture in Intestinal Organoids. Adv. Healthc. Mater..

[247] Pérez-González C., Ceada G., Greco F., Matejčić M., Gómez-González M., Castro N., Menendez A., Kale S., Krndija D., Clark A.G. (2021). Mechanical compartmentalization of the intestinal organoid enables crypt folding and collective cell migration. Nat. Cell Biol..

[248] He S., Lei P., Kang W., Cheung P., Xu T., Mana M., Park C.Y., Wang H., Imada S., Russell J.O. (2023). Stiffness Restricts the Stemness of the Intestinal Stem Cells and Skews Their Differentiation Toward Goblet Cells. Gastroenterology.

[249] Sorrentino G., Rezakhani S., Yildiz E., Nuciforo S., Heim M.H., Lutolf M.P., Schoonjans K. (2020). Mechano-modulatory synthetic niches for liver organoid derivation. Nat. Commun..

[250] Na J., Yang Z., Shi Q., Li C., Liu Y., Song Y., Li X., Zheng L., Fan Y. (2024). Extracellular matrix stiffness as an energy metabolism regulator drives osteogenic differentiation in mesenchymal stem cells. Bioact. Mater..

[251] Na J., Tai C., Wang Z., Yang Z., Chen X., Zhang J., Zheng L., Fan Y. (2025). Stiff extracellular matrix drives the differentiation of mesenchymal stem cells toward osteogenesis by the multiscale 3D genome reorganization. Biomaterials.

[252] Zhang C., Shen Y., Huang M., Wang G., Miao Q., Shi H., Gao R., Wang K., Luo M. (2026). Dynamic hydrogel mechanics in organoid engineering: From matrix design to translational paradigms. Bioact. Mater..

[253] Gan Z., Qin X., Liu H., Liu J., Qin J. (2023). Recent advances in defined hydrogels in organoid research. Bioact. Mater..

[254] Chaudhuri O., Cooper-White J., Janmey P.A., Mooney D.J., Shenoy V.B. (2020). Effects of extracellular matrix viscoelasticity on cellular behaviour. Nature.

[255] Sampayo R.G., Sakamoto M., Wang M., Kumar S., Schaffer D.V. (2023). Mechanosensitive stem cell fate choice is instructed by dynamic fluctuations in activation of Rho GTPases. Proc. Natl. Acad. Sci. USA.

[256] Wu Y., Song Y., Soto J., Hoffman T., Lin X., Zhang A., Chen S., Massad R.N., Han X., Qi D. (2025). Viscoelastic extracellular matrix enhances epigenetic remodeling and cellular plasticity. Nat. Commun..

[257] Nerger B.A., Kashyap K., Deveney B.T., Lou J., Hanan B.F., Liu Q., Khalil A., Lungjangwa T., Cheriyan M., Gupta A. (2024). Tuning porosity of macroporous hydrogels enables rapid rates of stress relaxation and promotes cell expansion and migration. Proc. Natl. Acad. Sci. USA.

[258] Crandell P., Stowers R. (2023). Spatial and Temporal Control of 3D Hydrogel Viscoelasticity through Phototuning. ACS Biomater. Sci. Eng..

[259] Courbot O., Elosegui-Artola A. (2025). The role of extracellular matrix viscoelasticity in development and disease. npj Biol. Phys. Mech..

[260] Yavitt F.M., Kirkpatrick B.E., Blatchley M.R., Speckl K.F., Mohagheghian E., Moldovan R., Wang N., Dempsey P.J., Anseth K.S. (2023). In situ modulation of intestinal organoid epithelial curvature through photoinduced viscoelasticity directs crypt morphogenesis. Sci. Adv..

[261] Peng Y.H., Hsiao S.K., Gupta K., Ruland A., Auernhammer G.K., Maitz M.F., Boye S., Lattner J., Gerri C., Honigmann A. (2023). Dynamic matrices with DNA-encoded viscoelasticity for cell and organoid culture. Nat. Nanotechnol..

[262] Chaudhuri O., Gu L., Klumpers D., Darnell M., Bencherif S.A., Weaver J.C., Huebsch N., Lee H.-P., Lippens E., Duda G.N. (2016). Hydrogels with tunable stress relaxation regulate stem cell fate and activity. Nat. Mater..

[263] O’conor C.J., Leddy H.A., Benefield H.C., Liedtke W.B., Guilak F. (2014). TRPV4-mediated mechanotransduction regulates the metabolic response of chondrocytes to dynamic loading. Proc. Natl. Acad. Sci. USA.

[264] Nam S., Gupta V.K., Lee H.-P., Lee J.Y., Wisdom K.M., Varma S., Flaum E.M., Davis C., West R.B., Chaudhuri O. (2019). Cell cycle progression in confining microenvironments is regulated by a growth-responsive TRPV4-PI3K/Akt-p27^Kip1^ signaling axis. Sci. Adv..

[265] Lee H.-P., Stowers R., Chaudhuri O. (2019). Volume expansion and TRPV4 activation regulate stem cell fate in three-dimensional microenvironments. Nat. Commun..

[266] Cavalcanti-Adam E.A., Volberg T., Micoulet A., Kessler H., Geiger B., Spatz J.P. (2007). Cell spreading and focal adhesion dynamics are regulated by spacing of integrin ligands. Biophys. J..

[267] Wang X., Yan C., Ye K., He Y., Li Z., Ding J. (2013). Effect of RGD nanospacing on differentiation of stem cells. Biomaterials.

[268] Kapp T.G., Rechenmacher F., Neubauer S., Maltsev O.V., Cavalcanti-Adam E.A., Zarka R., Reuning U., Notni J., Wester H.-J., Mas-Moruno C. (2017). A Comprehensive Evaluation of the Activity and Selectivity Profile of Ligands for RGD-binding Integrins. Sci. Rep..

[269] Lee S., Yoo J., Bae G., Thangam R., Heo J., Park J.Y., Choi H., Kim C., An J., Kim J. (2024). Photonic control of ligand nanospacing in self-assembly regulates stem cell fate. Bioact. Mater..

[270] Zhang X., Karagöz Z., Swapnasrita S., Habibovic P., Carlier A., van Rijt S. (2023). Development of Mesoporous Silica Nanoparticle-Based Films with Tunable Arginine–Glycine–Aspartate Peptide Global Density and Clustering Levels to Study Stem Cell Adhesion and Differentiation. ACS Appl. Mater. Interfaces.

[271] Zhang X., van Veen S., Hadavi D., Zhao Y., Mohren R., Habibović P., Honing M., Albertazzi L., van Rijt S. (2024). DNA Nanoparticle Based 2D Biointerface to Study the Effect of Dynamic RGD Presentation on Stem Cell Adhesion and Migration. Small.

[272] Page-McCaw A., Ewald A.J., Werb Z. (2007). Matrix metalloproteinases and the regulation of tissue remodelling. Nat. Rev. Mol. Cell Biol..

[273] Narasimhan B.N., Fraley S.I. (2025). Matrix degradation enhances stress relaxation, regulating cell adhesion and spreading. Proc. Natl. Acad. Sci. USA.

[274] Lutolf M.P., Lauer-Fields J.L., Schmoekel H.G., Metters A.T., Weber F.E., Fields G.B., Hubbell J.A. (2003). Synthetic matrix metalloproteinase-sensitive hydrogels for the conduction of tissue regeneration: Engineering cell-invasion characteristics. Proc. Natl. Acad. Sci. USA.

[275] Jhala D., Vasita R. (2015). A Review on Extracellular Matrix Mimicking Strategies for an Artificial Stem Cell Niche. Polym. Rev..

[276] Brusatin G., Panciera T., Gandin A., Citron A., Piccolo S. (2018). Biomaterials and engineered microenvironments to control YAP/TAZ-dependent cell behaviour. Nat. Mater..

[277] Rammensee S., Kang M.S., Georgiou K., Kumar S., Schaffer D.V. (2017). Dynamics of Mechanosensitive Neural Stem Cell Differentiation. Stem Cells.

[278] Madl C.M., Wang Y.X., Holbrook C.A., Su S., Shi X., Byfield F.J., Wicki G., Flaig I.A., Blau H.M. (2024). Hydrogel biomaterials that stiffen and soften on demand reveal that skeletal muscle stem cells harbor a mechanical memory. Proc. Natl. Acad. Sci. USA.

[279] Yang B., Wei K., Loebel C., Zhang K., Feng Q., Li R., Wong S.H.D., Xu X., Lau C., Chen X. (2021). Enhanced mechanosensing of cells in synthetic 3D matrix with controlled biophysical dynamics. Nat. Commun..

[280] Azagarsamy M.A., Anseth K.S. (2013). Bioorthogonal Click Chemistry: An Indispensable Tool to Create Multifaceted Cell Culture Scaffolds. ACS Macro Lett..

[281] Hui E., Sumey J.L., Caliari S.R. (2021). Click-functionalized hydrogel design for mechanobiology investigations. Mol. Syst. Des. Eng..

[282] Álvarez Z., Ortega J.A., Sato K., Sasselli I.R., Kolberg-Edelbrock A.N., Qiu R., Marshall K.A., Nguyen T.P., Smith C.S., Quinlan K.A. (2023). Artificial extracellular matrix scaffolds of mobile molecules enhance maturation of human stem cell-derived neurons. Cell Stem Cell.

[283] Majumder J., Torr E.E., Aisenbrey E.A., Lebakken C.S., Favreau P.F., Richards W.D., Yin Y., Chang Q., Murphy W.L. (2024). Human induced pluripotent stem cell-derived planar neural organoids assembled on synthetic hydrogels. J. Tissue Eng..

[284] Li Z., Li K., Zhang C., Zhao Y., Guo Y., He J., Chang S., Fang X., Liu K., Zhu P. (2025). Bioprinted Organoids: An Innovative Engine in Biomedicine. Adv. Sci..

[285] Li Z., Chen L., Wu J., Chen Y., Zhu Y., Li G., Xie G., Tang G., Xie M. (2025). A review of 3D bioprinting for organoids. Med Rev..

[286] Skylar-Scott M.A., Uzel S.G.M., Nam L.L., Ahrens J.H., Truby R.L., Damaraju S., Lewis J.A. (2019). Biomanufacturing of organ-specific tissues with high cellular density and embedded vascular channels. Sci. Adv..

[287] Stankey P.P., Kroll K.T., Ainscough A.J., Reynolds D.S., Elamine A., Fichtenkort B.T., Uzel S.G., Lewis J.A. (2024). Embedding Biomimetic Vascular Networks via Coaxial Sacrificial Writing into Functional Tissue. Adv. Mater..

[288] Bai L., Wu Y., Li G., Zhang W., Zhang H., Su J. (2024). AI-enabled organoids: Construction, analysis, and application. Bioact. Mater..

[289] Cadamuro F., Piazzoni M., Gamba E., Sonzogni B., Previdi F., Nicotra F., Ferramosca A., Russo L. (2025). Artificial Intelligence tool for prediction of ECM mimics hydrogel formulations via click chemistry. Biomater. Adv..

[290] Li Z., Song P., Li G., Han Y., Ren X., Bai L., Su J. (2024). AI energized hydrogel design, optimization and application in biomedicine. Mater. Today Bio.

[291] Zhang Z., Zhou X., Fang Y., Xiong Z., Zhang T. (2025). AI-driven 3D bioprinting for regenerative medicine: From bench to bedside. Bioact. Mater..

[292] Zhang Z., Wang Y., Wang W. (2025). Machine Learning in Gel-Based Additive Manufacturing: From Material Design to Process Optimization. Gels.

[293] Shi H., Kowalczewski A., Vu D., Liu X., Salekin A., Yang H., Ma Z. (2024). Organoid intelligence: Integration of organoid technology and artificial intelligence in the new era of in vitro models. Med. Nov. Technol. Devices.

[294] Zeevaert K., Mabrouk M.H.E., Wagner W., Goetzke R. (2020). Cell Mechanics in Embryoid Bodies. Cells.

[295] Pettinato G., Wen X., Zhang N. (2015). Engineering Strategies for the Formation of Embryoid Bodies from Human Pluripotent Stem Cells. Stem Cells Dev..

[296] Seitz M.P., Song Y., Lian X.L., Ma Z., Jain E. (2024). Soft Polyethylene Glycol Hydrogels Support Human PSC Pluripotency and Morphogenesis. ACS Biomater. Sci. Eng..

[297] Kim J.W., Nam S.A., Yi J., Kim J.Y., Lee J.Y., Park S.Y., Sen T., Choi Y.M., Lee J.Y., Kim H.L. (2022). Kidney Decellularized Extracellular Matrix Enhanced the Vascularization and Maturation of Human Kidney Organoids. Adv. Sci..

[298] Guo X., Liu B., Zhang Y., Cheong S., Xu T., Lu F., He Y. (2024). Decellularized extracellular matrix for organoid and engineered organ culture. J. Tissue Eng..

[299] Chrisnandy A., Lutolf M.P. (2025). An extracellular matrix niche secreted by epithelial cells drives intestinal organoid formation. Dev. Cell.

[300] Chen F., Zhang H.-Y., Wan Y.-L., Jia J.-N., Wang R.-Z., Gao C., Chao Z.-Y., Ru Y.-H., Wang Z., Cheng K. (2025). Artificial intelligence-assisted organoid construction in congenital heart disease: Current applications and future prospects. Front. Bioeng. Biotechnol..

[301] Moss S.P., Bakirci E., Feinberg A.W. (2025). Engineering the 3D structure of organoids. Stem Cell Rep..

[302] Zushin P.-J.H., Mukherjee S., Wu J.C. (2023). FDA Modernization Act 2.0: Transitioning beyond animal models with human cells, organoids, and AI/ML-based approaches. J. Clin. Investig..

